# Thermoresponsive Nanogels of Modified Poly((di(ethylene glycol) methyl ether methacrylate)-*co*-(2-aminoethyl methacrylate))s

**DOI:** 10.3390/polym12081645

**Published:** 2020-07-24

**Authors:** Daria Lipowska-Kur, Łukasz Otulakowski, Barbara Trzebicka, Alicja Utrata-Wesołek, Andrzej Dworak

**Affiliations:** Centre of Polymer and Carbon Materials, Polish Academy of Sciences, M. Curie-Sklodowskiej 34, 41-819 Zabrze, Poland; dlipowska@cmpw-pan.edu.pl (D.L.-K.); lotulakowski@cmpw-pan.edu.pl (Ł.O.); btrzebicka@cmpw-pan.edu.pl (B.T.); autrata@cmpw-pan.edu.pl (A.U.-W.)

**Keywords:** nanogels, thermoresponsive polymers, click chemistry, oligo(ethylene glycol) methacrylate

## Abstract

A series of copolymers of di(ethylene glycol) methyl ether methacrylate (D) and 2-aminoethyl methacrylate (A) (P(D-co-A)) with variable ratios of comonomers were synthesized using atom transfer radical polymerization. Then, the amino groups of obtained copolymers were modified to clickable azide or prop-2-yn-1-yl carbamate groups. A thermoresponsive copolymers were obtained with the value of cloud point temperature (T_CP_) dependent on the type and number of functional groups in the copolymer and on the concentration of solutions. For P(D-co-A) copolymers, the T_CP_ increased with increasing content of 2-aminoethyl methacrylate comonomer. The presence of azide and prop-2-yn-1-yl carbamate groups caused the changes of T_CP_ of modified copolymers. All studied copolymers in dilute aqueous solutions aggregated above T_CP_ to nanoparticles with sizes dependent on the solution concentration, heating procedures, and types and numbers of functional groups present in a copolymer chain. The presence of hydrophilic elements in the chain and the increase in the copolymer concentration led to the enlargement of the particle sizes. Aggregates were crosslinked using click reaction between an azide and prop-2-yn-1-yl carbamate groups that led to stable thermoresponsive nanogels. A systematic study of the behavior of copolymers allowed the determination of the chains useful for possible application in drug delivery.

## 1. Introduction

Polymeric nanogels (also called nano-sized hydrogels or hydrogel nanoparticles) are promising and innovative materials that have great potential for nanomedicine, pharmaceutics, and bio-nanotechnology [1,2,3]. Nanogels are three-dimensional networks that tend to adsorb water or physiological fluid without a change in their internal structure. They can be used as biosensors, cell culture systems, and recently quite often as drug delivery systems [1,4,5,6,7,8,9]. They have high stability, drug loading ability, biologic consistency, and can be responsive to environmental stimuli. They can protect the encapsulated biological molecules from degradation and body clearance and actively contribute to the delivery process to the target site.

The three-dimensional network of nanogels can be formed via chemical crosslinking (by covalent bonds) or via physical crosslinking (weaker linkages by non-covalent bonds). Several methods for nanogels preparation have been developed. Nanogels can be obtained by free-radical crosslinking polymerization, conventional and controlled/living radical polymerization techniques, photolithographic techniques, and by involving the polymeric precursors with the use of click chemistry, Schiff-base reactions, thiol-disulfide exchange, amide crosslinking, photo-induced crosslinking, or enzyme-mediated crosslinking [1,2,3].

One of the convenient ways for the preparation of nanogels is the crosslinking of spherical particles—mesoglobules that are formed by LCST-type thermoresponsive polymers. The thermoresponsive polymers undergo phase separation when polymer-polymer interactions start to dominate over chain interactions with water [10,11]. The polymers precipitate at characteristic temperature value, commonly known as cloud point temperature (T_CP_) [12,13]. This temperature can be easily controlled by the topology of macromolecules (linear, cyclic, branched, star polymers) [14,15,16,17,18], the molar mass of the polymer and its dispersion [19,20] and also by the presence of additives [21,22]. In the case of copolymers, the content and distribution (random, gradient, block) of hydrophilic and hydrophobic elements in the macromolecules are also essential [23,24,25]. The incorporation of hydrophilic monomer into the chain of a thermoresponsive polymer increases the T_CP_ value [10,25,26,27,28]. Conversely, the introduction of hydrophobic monomers into the polymer chain causes the T_CP_ value to decrease. The thermoresponsiveness can be even generated starting from non-thermoresponsive homopolymers by incorporation into their structure comonomers or functional groups with different affinity to water [23,29,30,31,32]. When the heating of thermoresponsive polymer is performed for a dilute solution, the process leads to the formation of mesoglobules with sizes from several dozen to several hundred nanometers [11,33]. The mesoglobule sizes are determined by the polymer concentration [34,35,36], molar mass [35], composition of the copolymer [24,37], or heating procedures of solutions [36,38].

So far, mesoglobules have been obtained from thermoresponsive polymers such as poly(*N*-vinyl caprolactam) [34], poly(methyl vinyl ether) [34], poly(*N*-isopropylacrylamide) [12,34,39] and its copolymers [37,40,41], poly(ethoxytriethyleneglycol acrylate) [42], poly(diethylene glycol) methacrylates [35] and its copolymers [25,36,43], *N,N*-diethylacrylamide copolymers [28], and oligo(ethylene glycol) and glycidol dendrimers [44,45].

The process of mesoglobule formation is reversible, and after lowering the temperature, the particles disaggregate. For potential applications, such behavior is not always desirable, at least in the initial period of material functioning. Therefore, mesoglobules stabilization by covering them with crosslinked shell formed during nucleated polymerization [24,46,47,48,49,50,51,52] or by the cross-linking of the collapsed polymer chains in a Huisgen reaction [25,43] has been performed.

In our previous works [25], we have shown that the poly((di(ethylene glycol) methyl ether methacrylate)-co-((2-aminoethyl) methacrylate)) (P(D-co-A)) with 8% of A groups is a thermoresponsive copolymer able for modification to the azide and prop-2-yn-1-yl carbamates groups. The resultant copolymers formed mesoglobules with potential use as drug nanocarriers [43]. In this article, we present a systematic study of a set of P(D-co-A) copolymers, and the products of their modification, focusing on determining in detail the impact of the copolymer composition and the type of functional groups on thermoresponsiveness of the copolymers and their behavior in aqueous solutions. The formation of thermoresponsive nanogels by the coaggregation of different pairs of the copolymers and the subsequent chemical crosslinking of obtained mesoglobules was followed. Efforts were made to determine the impact of the chain structure on the physico-chemical properties of obtained nanomaterials - response to temperature, swelling, availability of azide groups for potential post-modifications, sizes of aggregates, which has not been studied previously. The dependences established in this work should allow for selection of polymers optimal for the creation of nanocarriers.

## 2. Materials and Methods 

### 2.1. Materials

Di(ethylene glycol) methyl ether methacrylate (95%, D), copper (I) chloride (98%, CuCl), ethyl α-bromoisobutyrate (EBB, 98%), propargyl chloroformate (96%), 4-dimethylaminopyridine (99%, DMAP), 2-azido-1,3-dimethylimidazoliniumhexafluoro phosphate (97%, ADMP), and triethylamine (98%, TEA) were purchased from Sigma-Aldrich (Poznan, Poland). The 2-aminoethyl methacrylate hydrochloride (>98%, A) was received from Polysciences Inc. (Warrington, PA, USA). The 2,2′-bipyridine (99%, Bpy) was purchased from Alfa Aesar (Karlsruhe, Germany). Methyl alcohol (99.8%), ethyl alcohol (98%), dichloromethane (99.8%, DCM), *N*,*N*-dimethylformamide (DMF), and tetrahydrofuran (99.5%, THF) were purchased from Avantor™ Performance Materials (Gliwice, Poland). The DCM and DMF were distilled before use. The THF was distilled over potassium hydroxide before use. The water used to prepare polymer solutions was purified using a commercial ion exchange system (Hydrolab Company, Straszyn, Poland). The polymer solutions were kept overnight at 8 °C before use. Other reagents were used as received.

### 2.2. Synthesis of Di(ethylene glycol) Methyl Ether Methacrylate (D) and 2-aminoethyl Methacrylate Hydrochloride (A) Copolymers

Synthesis of P(D-co-A) was performed similarly to a procedure previously described by us [25]. As an example, the representative synthesis of the P(D-co-A) copolymer with 3% of A content and its subsequent modifications with prop-2-yn-1-yl carbamate and azide groups are presented below.

Monomers (D, 5.29 mL, 28.10 mmol; A, 0.405 g, 2.44 mmol), a ligand (Bpy, 0.032 g, 0.204 mmol), a catalyst (CuCl, 0.01 g, 0.102 mmol), and a 7.8 mL methanol/water mixture (2:1 v/v) were placed in a Schlenk flask equipped with a magnetic stirrer and an argon/vacuum inlet valve. The solution was deoxygenated by three freeze–vacuum–thaw cycles. After the second cycle, the initiator (EBB, 15 μL, 0.102 mmol) was introduced into the system under an argon atmosphere. For all reactions, the initial ratio of EBB/CuCl/Bpy was 1/1/2. The polymerization was carried out for 24 h at room temperature and was finished by purging the system with air. The copolymer was purified by dialysis through a membrane (cut off 3500 Da) against acetone for 5 days. The obtained copolymers were denoted as P(D-co-A)_1, P(D-co-A)_2, P(D-co-A)_3, P(D-co-A)_4, and P(D-co-A)_5 with respect to increasing A content.

### 2.3. Synthesis of Prop-2-yn-1-yl Carbamate Modified Copolymers

Modification of amine groups in poly((di(ethylene glycol) methyl ether methacrylate)-co-(2-aminoethyl methacrylate)) with propargyl chloroformate was carried out in accordance to the procedure described in [25]. First, P(D-co-A)_1 (0.313 g, 0.0105 mmol) and triethylamine (0.353 mL, 2.52 mmol) were dissolved in 2 mL of THF. The polymer solution was placed in an ice bath and propargyl chloroformate (0.244 mL, 2.52 mmol) was added. After one hour, the reaction mixture was moved to room temperature and left overnight. The poly((di(ethylene glycol) methyl ether methacrylate)-co-(2-N-(prop-2-yn-1-yl carbamate)ethyl methacrylate)) was purified by dialysis through a membrane (cut off 3500 Da) against water/acetone mixture for 1 day and next against pure acetone for 4 days. The resulting samples were denoted as P(D-co-Prop)_1, P(D-co-Prop)_2, P(D-co-Prop)_3, P(D-co-Prop)_4, and P(D-co-Prop)_5, where numbers 1 to 5 correspond to the respective P(D-co-A) copolymer that was modified.

### 2.4. Synthesis of Azide Modified Copolymers

The amine groups of poly((di(ethylene glycol) methyl ether methacrylate)-co-(2-aminoethyl methacrylate)) were converted to azides using a procedure described in [25]. P(D-co-A)_1 (0.200 g, 0.0067 mmol) and 4-dimethylaminopyridine (0.082 g, 0.671 mmol) were dissolved in 2 mL of dichloromethane. Then, 2-azido-1,3-dimethylimidazolinium hexafluorophosphate (0.192 g, 0.673 mmol) was added and the reaction mixture was boiled under reflux for 5 hours. The obtained product was purified by dialysis through a membrane (cut off 3500 Da) against water/acetone mixture and next against pure acetone. The resulting copolymers poly((di(ethylene glycol) methyl ether methacrylate)-co-(2-azidoethylmethacrylate)) were denoted as P(D-co-N_3_)_1, P(D-co-N_3_)_2, P(D-co-N_3_)_3, P(D-co-N_3_)_4, and P(D-co-N_3_)_5, where numbers 1 to 5 correspond to the respective P(D-co-A) copolymer that was modified.

### 2.5. Determination of Amine Groups in Copolymers (Kaiser Test)

To calculate the number of free amine groups present in copolymer chains, a reaction with ninhydrin was performed. For that purpose, 100 μL of 5% ninhydrin/n-BuOH, 50 μL of 80% phenol/n-BuOH and 50 μL of 0.01 moll/L KCN/pyridine were added to polymer samples and the mixture was shortly heated to 100 °C. The absorbance of the post-reaction solutions in ethyl alcohol was measured by UV-Vis at 586 nm. 

### 2.6. Aggregation of Copolymers

Aggregation of P(D-co-A)_x, P(D-co-N_3_)_x, P(D-co-Prop)_x, and the binary mixtures of P(D-co-Prop)_x/P(D-co-N_3_)_x (1:1 w/w) were performed by gradual heating. The method involved gradual heating of 2 mL of the polymer solutions (c = 0.1, 0.2, 0.5, 1.0, 5.0, and 10.0 g/L) from 10 °C to 70 °C using a thermo-controller (the temperature increase occurred every 2–4 degrees with 10 minutes of stabilization).

### 2.7. Nanogels Formation

Nanogels were formed by aggregation of a binary mixture of copolymers containing azide and prop-2-yn-1-yl carbamate groups followed by subsequent crosslinking. Crosslinking was performed in water solution using the Huisgen 1,3-dipolar cycloaddition of azides and alkynes as described previously [25]. First, 4 mL of solutions of mixed P(D-co-Prop)_x/P(D-co-N_3_)_x copolymers (1:1 w/w) at 0.5 g/L were abruptly heated from 8 to 60 °C and crosslinked by addition of copper (II) sulfate pentahydrate dissolved in 10 μL of water and 10 μL of a water solution containing sodium ascorbate (1:10 molar ratio Cu/NaAsc). All reactions were performed for 4 h at 60 °C. Obtained nanoparticles were dialyzed against water. Afterwards, they were measured by DLS above and below the T_CP_ of the copolymers at 60 and 10 °C, respectively.

### 2.8. Proton Nuclear Magnetic Resonance (^1^H NMR)

^1^H NMR spectra of copolymers in CDCl_3_ were recorded on a Bruker™ Ultrashield 600 Plus spectrometer (Billerica, MA, USA) operating at 600 MHz with TMS as the reference.

### 2.9. Fourier Transform Infrared Spectroscopy (FTIR)

The infrared spectra of copolymers films deposited on a KBr crystal were obtained using a Nicolet FTIR 6700 spectrometer (ThermoFisher Scintific, Madison, WI, USA) working in the transmission mode. All spectra were acquired between 4000 and 450 cm^−1^ using 124 scans and a 4 cm^−1^ resolution. The spectra were evaluated using OMNIC™ software (ThermoFisher Scintific, Madison, WI, USA).

### 2.10. Gel Permeation Chromatography (GPC-MALLS)

Gel permeation chromatography with a Dn-2010 RI differential refractive index detector (WGE Dr. Bures, Berlin, Germany) and a multiangle laser light scattering detector (DAWN EOS from Wyatt Technologies, Santa Barbara, CA, USA) was used to determine the molar masses and molar mass dispersities of the copolymers. The GPC was performed in DMF at 45 °C and at a nominal flow rate of 1 mL/min using the following set of columns: Polymer Standard Service (PSS) GRAM 100 Å, PSS GRAM 1000 Å, PSS GRAM 3000 Å. The refractive index increments of copolymers were independently measured as a batch method for 6 copolymer solutions in DMF at concentration from 0.5 g/L to 8 g/L using a SEC-3010 differential refractive index detector (WGE Dr. Bures, Berlin, Germany) and were used to calculate the average molar masses of the copolymers. The results were evaluated using ASTRA 5 software (Wyatt Technologies, Santa Barbara, CA, USA).

### 2.11. High-Performance Reverse-Phase Liquid Chromatography (RP-HPLC)

The progress of polymerizations was followed by RP-HPLC in the inverted phase system. The chromatographic system (Agilent, 1260 Infinity, Santa Clara, CA, USA) equipped with an Eclipse XDB-C18 column (4.6 × 150 mm, Agilent) and a UV-Vis detector (diode array, Agilent, 1260 DAD VL) was used for analyses. Analyses were performed in a 10 to 95% B mobile phase gradient system for 25 minutes (A: 0.1% TFA in water; B: 0.1% TFA in ACN). Chromatograms were recorded at 220 nm using Agilent ChemStation software (Santa Clara, CA, USA).

### 2.12. Cloud Point Measurement

The cloud point temperatures of the copolymers in aqueous solution were determined using a SPECORD 200 PLUS UV–Vis spectrophotometer from Analytik Jena (Jena, Germany) with a Peltier temperature-controlled cell holder (Analytik Jena, Jena, Germany) The transmittance was monitored as a function of temperature at λ = 550 nm for copolymer concentrations of 0.1, 0.2, 0.5, 1.0, 5.0, and 10 g/L. Solutions were heated gradually with a 1 °C step to the final temperature with a precision of 0.5 °C. The stabilization time after reaching the desired temperature was 60 s. The cloud points were referred as the inflection points of the transmittance curves.

### 2.13. Dynamic Light Scattering (DLS)

DLS measurements were performed on a Brookhaven BI-200 goniometer (Brookhaven Instruments, New York, NY, USA) with a vertically polarized incident light of wavelength λ = 637 nm supplied by a semiconductor laser diode operating at 36 mW and equipped with a Brookhaven BI-9000 (Brookhaven Instruments, New York, NY, USA) AT digital autocorrelator. To determine the thermoresponsiveness of the copolymers, the scattered light was measured at an angle of 90° for aqueous copolymers solutions at concentrations of 0.1, 0.2, 0.5, 1.0, 5.0, and 10.0 g/L from 8–70 °C. The cloud point temperatures were referred to the inflection points of the dependence of the R_h_^90^ versus temperature. The mesoglobules sizes (formed by P(D-co-A)_x, P(D-co-N_3_)_x, P(D-co-Prop)_x, and the binary mixtures of P(D-co-Prop)_x/P(D-co-N_3_)_x) were read for temperatures above the transition temperature. The R_h_^90^ measurements for nanogels were performed above and below the T_CP_ of the copolymers at 60 and 10 °C, respectively. The autocorrelation functions were analyzed by the constrained regularized algorithm CONTIN to obtain distributions of relaxation rates (Γ). The latter provided distributions of the apparent diffusion coefficient (D = Γ/q2), where q is the magnitude of the scattering vector, q = (4πn/λ)sin(θ/2) and n is the refractive index of the medium. The apparent hydrodynamic radius (R_h_^90^) was obtained from the Stokes–Einstein Equation (1)

R_h_^90^ = kT/6πηD (1)

for θ = 90° where k is the Boltzmann constant, η is the viscosity of water at temperature T, and D is the apparent diffusion coefficient. The dispersity of particle sizes (PDI) was given as μ2/Γ^−2^, where Γ^−^ is the average relaxation rate and μ2 is its second moment. 

### 2.14. Atomic Force Microscopy (AFM)

AFM measurements were performed using MultiMode with a NanoScope 3d controller (di-Veeco Instruments Inc., CA, USA), which was operated in the tapping mode in the air with standard 125-mm single-crystal silicon cantilevers (Model TESP, BRUKER, Billerica, MA, USA). Images were obtained using a piezoelectric scanner with a nominal size of 10 × 10 μm. Micrographs were recorded using NanoScope Software V531r1 (di-Veeco Instruments Inc., CA, USA). A 10-μL sample of nanogel water solution at a 0.5 g/L concentration was dropped on a mica slide and spin-coated for 1 h with a rotation speed of 400 rpm. The most representative images for each sample were selected from three measurements at different surface objects.

### 2.15. Cryogenic Transmission Electron Microscopy (CryoTEM)

Cryo-TEM images were obtained using a Tecnai F20 X TWIN microscope (FEI Company, Hillsboro, OR, USA) equipped with field emission gun, operating at an acceleration voltage of 200 kV. Images were recorded on the Gatan Rio 16 CMOS camera (Gatan Inc., Pleasanton, CA, USA) and processed with Gatan Microscopy Suite (GMS) software (Gatan Inc., Pleasanton, CA, USA). Specimen preparation was done by vitrification of the aqueous solutions on grids with holey carbon film (Quantifoil R 2/2; Quantifoil Micro Tools GmbH, Großlöbichau, Germany). Before use, the grids were activated for 15 seconds in oxygen plasma using a Femto plasma cleaner (Diener Electronic, Ebhausen, Germany). Cryo-samples were prepared by applying a droplet (3 μL) of the suspension to the grid, blotting with filter paper and immediate freezing in liquid ethane using a fully automated Vitrobot Mark IV blotting device (Thermo Fisher Scientific, Waltham, MA, USA). After preparation, the vitrified specimens were kept under liquid nitrogen until they were inserted into a Gatan 626 cryoTEM holder (Gatan Inc., Pleasanton, CA, USA) and analyzed in the TEM at −178 °C.

## 3. Results and Discussion

### 3.1. Synthesis and Characterization of P(D-co-A) Copolymers and Their Modified Derivatives

**Figure 1:**
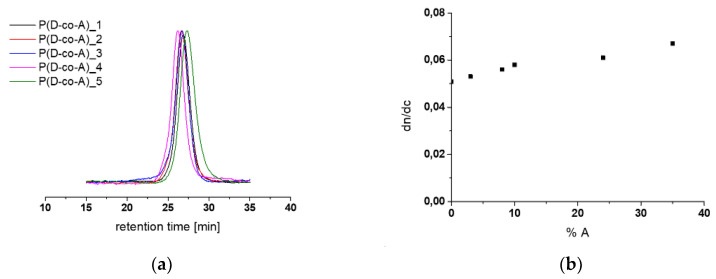
(**a**) GPC traces of copolymers (DMF, 1 mL/min); (**b**) the refractive index increments of P(D-co-A)_x copolymers (dn/dc) as a function of the amount of A in copolymer chain.

A series of syntheses of copolymers of di(ethylene glycol) methyl ether methacrylate (D) and 2-aminoethyl methacrylate hydrochloride (A) with increasing content of A were carried out. The syntheses were performed using atom transfer radical polymerization (ATRP) according to the procedure described in [25] (Figure S1a). Five copolymers, P(D-co-A)_1 up to P(D-co-A)_5, were obtained. Their molar masses and molar mass dispersities measured using gel permeation chromatography with multiangle light scattering detection (GPC-MALLS) are given in Table 1. The chromatograms are shown in Figure 1a.

As can be seen, the chromatograms were monomodal and narrow for each copolymer obtained (Figure 1a). For each synthesis, the conversion of the D and A units, measured via HPLC, reached no more than 50 ± 10%. The number average molar masses were in the range of 30,500–41,000 g/mol and molar mass dispersities (M_w_/M_n_) were less than 1.2 (Table 1). As can be seen in Table 1, the control of the molar masses was satisfactory for some copolymers. For molar mass calculation, the refractive index increments (dn/dc) for each copolymer were independently measured. As can be seen in Figure 1b, the dn/dc indexes of copolymers increased with the increasing content of A in the copolymer.

**Figure 2:**
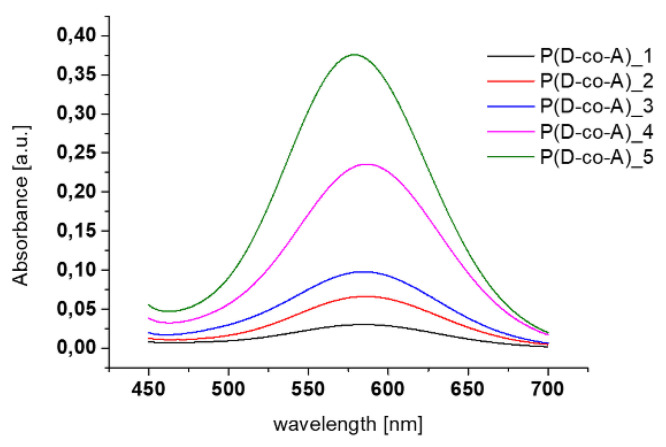
Absorbance of P(D-co-A) copolymers before modifications.

The quantity of A unit in the chains was determined using a ninhydrin reaction (the Kaiser test). The absorbance of P(D-co-A) copolymer solutions measured by UV-Vis at 586 nm (Figure 2) was used to calculate the content of free amine groups (Table 1). As can be noticed, the increase in the amount of A in the polymerization mixture resulted in A content increase in the copolymer chain.

Amino groups of all of the obtained P(D-co-A) copolymers were subsequently modified to clickable prop-2-yn-1-yl carbamate (Prop) groups or azide (N_3_) according to the methodology described previously [25]. Such a procedure ensures 100% of the substitution of amine groups. For that purpose, amine groups were reacted with propargyl chloroformate, which led to prop-2-yn-1-yl groups with a terminal alkyne, or with 2-azido-1,3-dimethyl imidazolinium hexafluorophosphate, which led to azide functions in the copolymer chains (Figure S1b,c).

As confirmed by the Kaiser test (Figure S2), the quantitative conversion of amino groups to azide and prop-2-yn-1-yl carbamate was achieved. In absorbance measurements, no absorption at 586 nm for copolymers with azide and prop-2-yn-1-ylcarbamate group was observed in contrary to absorption visible for copolymers with amine groups.

The NMR and FTIR analysis were performed to confirm the structure of P(D-co-A) copolymers and their modified derivatives with azides groups (P(D-co-N_3_)) and prop-2-yn-1-yl carbamate groups (P(D-co-Prop)) (Figures S3 and S4). The successful introduction of these functional groups into the copolymer chain led to copolymers capable for click reaction and nanogel formation.

### 3.2. Thermoresponsiveness of Copolymers

The main aim of this study was the systematic research focusing on determining the influence of the amount and kind of functional groups (amine, azide, or prop-2-yn-1-yl carbamate) on the behavior of so obtained copolymers and the pairs of respective copolymers in aqueous solution (thermoresponsiveness, the possibility of nanoparticle formation) and on determining the optimal composition of the copolymer to produce stable nanogels of controlled parameters.

**Figure 3:**
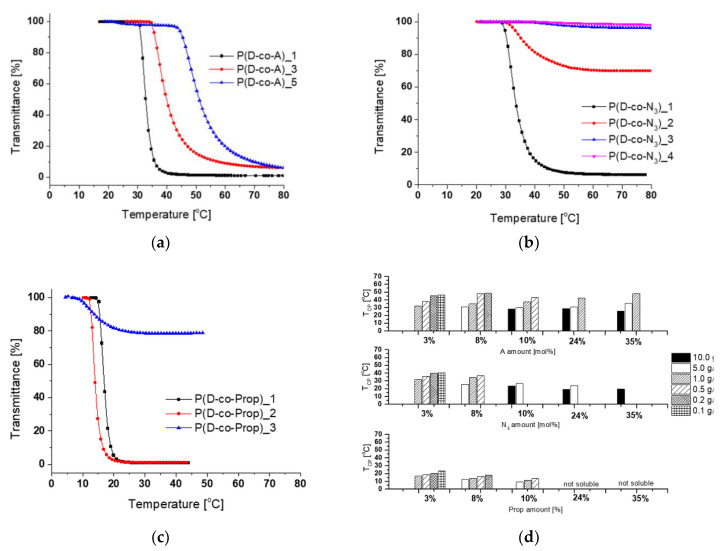
Transmittance versus temperature for (**a**) P(D-co-A)_1, P(D-co-A)_3, P(D-co-A)_5, (**b**) P(D-co-N_3_)_1, P(D-co-N_3_)_2, P(D-co-N_3_)_3, P(D-co-N_3_)_4, (**c**) P(D-co-Prop)_1, P(D-co-Prop)_2, P(D-co-Prop)_3 in water at a concentration of 1.0 g/L and (**d**) summary of phase transition temperatures for concentrations of 10.0, 5.0, 1.0, 0.5, 0.2, and 0.1 g/L of all polymers before and after modifications.

The thermoresponsive behavior of the set of P(D-co-A) copolymers and their derivatives qualitative modified with azide or propargyl groups was studied in dependence on copolymer composition and concentration in water using UV-Vis and DLS measurements. In Table 2 and Figure 3, the T_CP_ values for all copolymers are presented for the chosen solution concentration of 1.0 g/L (for the results of the all solution concentrations see Table S1, Figures S5–S7).

For copolymers P(D-co-A) before modification of the amine groups in the copolymer chain, the transition temperatures (for a certain copolymer concentration) increased with an increase of the amount of comonomer A (Table S1, Figure 3a). This confirms the hydrophilic nature of 2-aminoethyl methacrylate groups. For example, the T_CP_ of P(D-co-A)_1 with only 3% of amine groups was found at 32.2 °C, but for P(D-co-A)_5, which contains 35% of amine groups, the higher hydrophilicity of the copolymer shifted the T_CP_ almost of 16 degrees, to a temperature at 48.0 °C (Table 2). The T_CP_ values for P(D-co-A) copolymers (for a certain A content) increased with the decrease of the copolymer concentration (Table S1, Figure 3d), which has been observed for other thermoresponsive polymers [24,36,53]. With an increase in the number of amine groups in the copolymer chain, the T_CP_ could be distinguished on transmittance curves only for higher copolymer concentrations (Figure S5). For P(D-co-A)_1 (3% of amine groups) it was possible to detect the T_CP_ already from the 0.1 g/L concentration, but for P(D-co-A)_5 (35% of amine groups) the T_CP_ was measurable only for more concentrated solutions (0.5 g/L to 10.0 g/L) (Figure 3d).

Modification of the amine groups into azide groups and prop-2-yn-1-yl carbamate groups led to the decrease of T_CP_ of the resulting P(D-co-N_3_) and P(D-co-Prop) copolymers (Table 2 and Table S1). For the P(D-co-N_3_) series, the decrease in T_CP_ relative to the T_CP_ of the initial copolymer was not significant. For a certain copolymer concentration, the more azide groups in the copolymer, the higher the T_CP_ (Figure 3b). For all copolymers from a P(D-co-N_3_) series, a lower polymer concentration resulted in a weaker polymer reaction to temperature changes (Figure S6). For copolymers P(D-co-N_3_)_2 and P(D-co-N_3_)_3 (8 and 10% of azide groups) at the lowest concentrations, the transmittance dropped to only 96%, making the determination of T_CP_ impossible. The formation of particles, however, was detected via DLS measurement, although the values of scattering intensity (cps) were significantly lower in comparison to other measured concentrations. This indicated that these polymers are also thermoresponsive at these conditions. Nevertheless, for the P(D-co-N_3_) series, a low number of azide groups and high copolymer concentrations is necessary to induce clear temperature responsiveness (Figure 3d).

The incorporation of prop-2-yn-1-yl carbamate groups into the copolymer chain significantly reduced the T_CP_ of the resulting copolymer for each concentration (Table 2, Table S1). It can be also seen that the higher the content of prop-2-yn-1-yl carbamate groups in the copolymer, the lower is its T_CP_ (Figure 3c). The solutions of P(D-co-Prop)_4 and P(D-co-Prop)_5 copolymers (the 24 and 35% functional propargyl groups) were cloudy around 4 °C, and their T_CP_ could not be determined. For a certain number of propargyl groups, the increase in P(D-co-Prop) concentration resulted in a decrease of T_CP_ (Figure 3d, Figure S7).

The values of transition temperatures of all copolymers determined with the use of dynamic light scattering during gradual heating of their solutions slightly differed from those obtained from UV-Vis measurements, but the tendency reflecting the impact of the type of functional groups, their amount and copolymer concentration was preserved (Table 2, Table S1).

The studies of the influence of the structure and concentration of the P(D-co-A), P(D-co-N_3_), and P(D-co-Prop) copolymers on their cloud point temperature were necessary to select the conditions for obtaining mesoglobules and their crosslinking used in further research.

### 3.3. Aggregation of Thermoresponsive Copolymers

**Figure 4:**
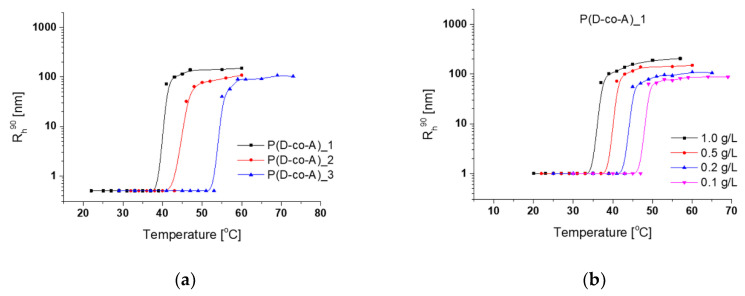

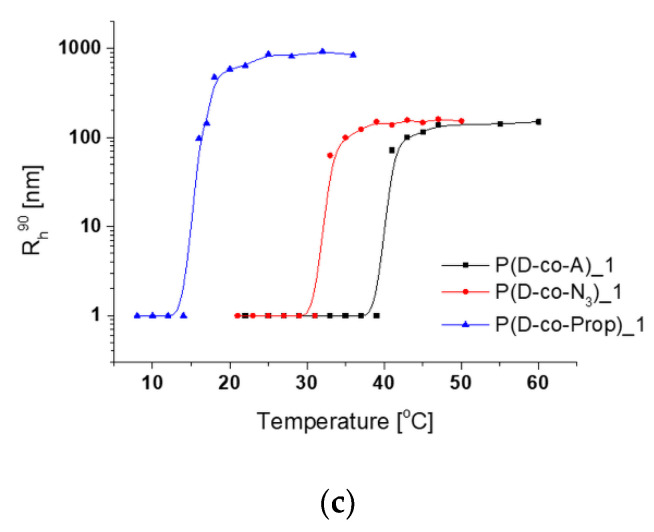
Apparent hydrodynamic radius ($R_{h}^{90}$) of particles versus temperature for (**a**) P(D-co-A)s at a concentration of 0.5 g/L; (**b**) P(D-co-A)_1 at different solution concentrations; (**c**) P(D-co-A)_1, P(D-co-N3)_1, and P(D-co-Prop)_1 at a concentration of 0.5 g/L.

As discussed in the Introduction, thermoresponsive polymers above phase transition temperature in a dilute water solution form spherical particles called mesoglobules. The sizes of these particles are determined by the solution concentration [34,36], the composition of the (co)polymer [36,37], and the heating rate of the solution [24,25,36]. The aggregation of P(D-co-A), P(D-co-N_3_), and P(D-co-Prop) chains was followed by DLS during gradual heating of copolymer solutions (see the Materials and Methods section). Individual polymer chains aggregated in a narrow temperature range (Figure 4). The sizes of particles formed by the studied copolymers at 0.5 g/L solution concentrations are shown in Table 3 (for the results of all solution concentrations see Table S2). Above the phase transition, the sizes of nanoparticles were stable with further heating of dispersions. The average dispersity (PDI) values were 0.122, 0.074, and 0.106 for mesoglobules obtained from P(D-co-A), P(D-co-N_3_), and P(D-co-Prop), respectively.

The presence of hydrophilic amino groups in the P(D-co-A) resulted in mesoglobules with sizes of 43–150 nm depending on the amine content in the copolymer and on the copolymer concentration (Table 3 and Table S2). The P(D-co-A) mesoglobules sizes were the same or smaller as compared to those obtained in similar conditions from respective P(D-co-N_3_) and P(D-co-Prop). During mesoglobule formation, the amine groups are probably predominantly concentrated on the surface of the collapsed particle, reducing the contact of the aggregates and thus the size of nanostructures. This assumption can also be confirmed by the fact that for a certain concentration an increase in the number of amine groups in the copolymer chain caused the decrease of the aggregate sizes (Table 3). The same tendency was observed for P(D-co-N_3_). For copolymers with the highest amount of amine and azide groups (24 mol% and 35 mol%), for a concentration of 0.5 g/L, the chain to globule transition may occur, but the globules of single chains are too small to be detected by DLS as well to cause transmittance changes (Table S1, Figures S5 and S6). Similar phenomena have been observed for poly(*N*-isopropylacrylamide)-g-poly(ethylene oxide) copolymers [54]. P(D-co-A) and P(D-co-N_3_) copolymers form well-defined mesoglobules when a lower number of functional groups are present in the chain, or a higher concentration is used. 

The introduction of 3 and 8% of hydrophobic propargyl groups into the copolymer chain led to particles with sizes up to 950 nm. The sizes are significantly larger than those obtained for their amine or azide counterparts (Table 3, Table S2). Only for the P(D-co-Prop)_3 copolymer with 10% of functional groups particles with sizes comparable to their amine or azide counterparts were formed. Similar observations were noticed in [24] where more hydrophobic poly(glycidol-co-ethyl glycidyl carbamate) with 35% of carbamate groups formed smaller mesoglobules than those formed by poly(glycidol-co-ethyl glycidyl carbamate) with 28% of carbamate groups. Here, the heating of thermoresponsive copolymer with a high amount of hydrophobic elements probably led to their concentration and “tightening”, resulting in aggregate shrinking inside the mesoglobules. 

It is known that the concentration of polymer solutions significantly affects the size of the resulting mesoglobules [24,25,34]. With a decrease in polymer concentration, the intra-chain shrinking of macromolecules dominates over the formation of inter-chain connections, which results in a decrease in the size of the aggregates formed. This dependence can be seen for all studied copolymers in Table S2. 

The results discussed above indicate that the sizes of mesoglobules can be controlled by the type of the functional groups, their number in the copolymer chain, and the copolymer concentration. As is known [49,50], the sizes of thermally aggregated particles can also be influenced by the heating rate of the polymer solution. The gradual heating that has been applied in this work allowed the tracking of the thermoresponsiveness of the copolymers but led to the formation of relatively large nanostructures with sizes up to 950 nm. The abrupt heating of thermoresponsive polymer solutions is characterized by the formation of particles of much smaller sizes than with gradual heating [36,42,49,50]. 

### 3.4. Nanogel Formation

As we have shown previously [25], an easy procedure to create stable crosslinked nanoparticles is to explore the formation of mesoglobules in a solution of mixed thermoresponsive copolymers during abrupt heating. This procedure led to the formation of homogenously co-mixed aggregated particles, even if the difference in transition temperature of particular copolymers reached up to 20 °C [25,50]. That is why, in this work, we used the abrupt heating procedure to obtain crosslinked, thermoresponsive spherical nanogels of P(D-co-N_3_) and P(D-co-Prop) copolymers. The impact of the number of functional groups in the chain on the efficient crosslinking and the stability of formed particles was studied. 

Nanogels were obtained by aggregation of pairs of copolymers containing azide and prop-2-yn-1-yl carbamate functional groups, at a 1:1 w/w copolymer content. The aggregation was performed at 60 °C at a total concentration equal to 0.5 g/L. The aggregation was followed by Huisgen 1,3-dipolar cycloaddition (See Materials and Method Section) between copolymers. The reaction proceeds under mild conditions with high selectivity and efficiency, which makes it very attractive for the stabilization of polymeric nanoparticles [55].

Mixed systems were obtained using pairs of copolymers: P(D-co-N_3_)_1 with P(D-co-Prop)_1 (R1), P(D-co-N_3_)_2 with P(D-co-Prop)_2 (R2), and P(D-co-N_3_)_3 with P(D-co-Prop)_3 (R3), having 5, 17, and 21 reactive functional groups in each copolymer chain (Table 1), respectively. The systems that possessed different numbers of reactive azide and propargyl groups were also studied. These were P(D-co-N_3_)_3 with P(D-co-Prop)_2 (R4), P(D-co-N_3_)_2 with P(D-co-Prop)_1 (R5), and P(D-co-N_3_)_3 with P(D-co-Prop)_1 (R6), where the 4, 12, and 16 free azide groups, respectively should remain unreacted after crosslinking. Here, the effect in the difference in the amount of crosslinkable groups in mixed copolymers on the stability and swelling of their nanogels was followed.

**Figure 5:**
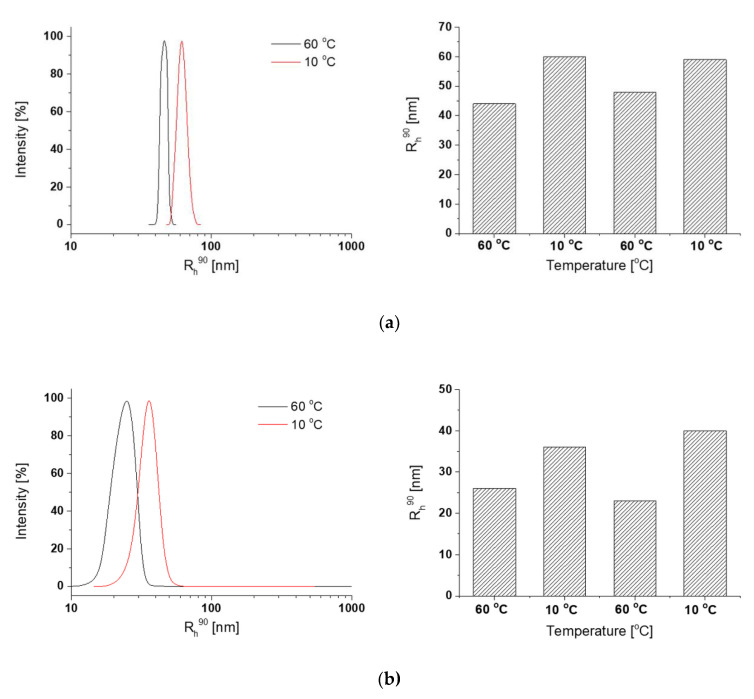
Exemplary size distributions of the crosslinked nanoparticles formed in aqueous solutions and the changes of their radii during heating and cooling cycles at total concentration 0.5 g/L of (**a**) P(D-co-N_3_)_1/P(D-co-Prop)_1 (R1) and (**b**) P(D-co-N_3_)_3/P(D-co-Prop)_2 (R4) (1:1).

The DLS size distributions of crosslinked nanoparticles, measured above and below the temperature transitions for the copolymers that form them, are shown in Figure 5 and Table 4. 

Crosslinking experiment results show the impact of the content of azide and prop-2-yn-1-yl carbamate groups on the sizes and swelling ratio of acquired nanoparticles (Table 4). In the cases of the R1, R2, R4, and R5 mixtures, single populations of particles before and after crosslinking were obtained. The crosslinking process did not influence the sizes of these nanogels formed by the chains; the $R_{h}^{90}$ before and after crosslinking are similar. The particles were stable during heating and cooling cycles (Figure 5). They reversibly changed their radii in response to abrupt temperature changes, which confirmed their thermoresponsivity. The difference in the R_h_ between 60 and 10 °C was about 10 to 20 nm.

When the same amount of azide and propargyl groups in the binary mixtures of copolymers was used for nanogel formation, no significant influence of the amount of these groups on the size of R1 and R2 nanogels was observed. However, as indicated by the swelling ratio, the nanogel R2 (with a larger amount of reactive groups) seemed to be more densely crosslinked than R1; thus water had weaker access to its particles (Table 4). 

The ratio of functional groups (azide versus propargyl) present in copolymers influenced nanoparticle properties. The nanogel R4 had 17 azide and 17 propargyl groups per chain that should be crosslinked and 4 azide groups per P(D-co-N_3_) chain that should remain uncrosslinked. These had lower $R_{h}^{90}$ values and slightly lower swelling ratios than the nanogel R5, where only 5 pairs of reactive groups should be crosslinked and 12 azide groups per chain should be unreacted (Table 4). This indicates that the R4 nanogel was more crosslinked, and due to a lower amount of free hydrophilic azide groups, it did not absorb a substantial amount of water.

The influence of free azide groups on the particle sizes and swelling behavior can be seen in the cases of nanogels R1 and R5. In both copolymer pair forming nanoparticles, the degree of crosslinking should be the same (5 azide and propargyl groups potentially applied for crosslinking), while in R5, twelve azides should remain unreacted. It can be seen that a slight increase in R_h_ and swelling ratio is observed with increasing free azides. This confirms the hydrophilic nature of this group that finally leads to loosely packed nanogels of high swelling capacity. A similar situation exists for nanogels R2 and R4 with 17 crosslinked groups. The free, uncrosslinked hydrophilic azide groups present in R4 nanogel caused a higher swelling ratio of the nanogel.

A slightly different situation, in comparison to nanogels described so far, can be observed for nanogels R3 and R6. In the case of R3 nanogels (for the formation of which copolymers with the highest amount of functional groups were used), two populations of particles were visible before and after crosslinking (Table 4, Figure S8). The small size population (26 nm before crosslinking and 42 nm after crosslinking) probably contains the mixture of P(D-co-N_3_)_3 with P(D-co-Prop)_3. The R_h_ of the particles formed separately by these copolymers measured during gradual heating exceeded 76 and 68 nm (Table 3). Thus, it could be expected [24,56] that abrupt heating of the R3 mixture would lead to a population with significantly smaller sizes and dense cores. The population of larger sizes could arise because of the crosslinking of adjacent small particles that possess functional groups concentrated on their surface. In the case of the R6 nanogel, characterized by the highest difference in the number of reactive functional groups used for crosslinking, the creation of nanogel was not obvious (Table 4, Figure S8). The population of small particles present before crosslinking could be formed by the P(D-co-N_3_)_3 copolymer, and the larger size population could be formed mainly by P(D-co-Prop)_1 (during gradual heating this copolymer formed particles of 599 nm). After crosslinking, one population with R_h_ of about 150 nm was formed. The structure was probably loosely crosslinked. The low swelling of the R6 nanogel may point to the fact that it is not composed of P(D-co-N_3_)_3 and P(D-co-Prop)_1 copolymers in equal proportions, but the hydrophobic P(D-co-Prop)_1 constitutes a major part.

**Figure 6:**
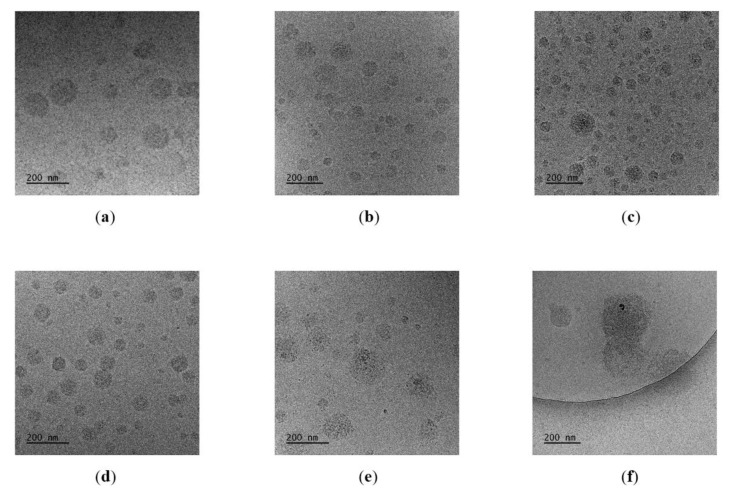
CryoTEM images of (**a**) R1, (**b**) R2, (**c**) R3, (**d**) R4, (**e**) R5, (**f**) R6 nanogels (C = 0.5 g/L).

CryoTEM images obtained in this work for crosslinked nanoparticles are shown in Figure 6. The histograms taking into account sizes of nanoparticles were constructed from cryoTEM images, and they are given in Figure S9.

The sizes of nanogels obtained via cryoTEM for R1, R2, R4, and R5 were comparable to those obtained by DLS. For the R3 nanogel images, two populations of particles were also visible with cryoTEM. The number of large particles was small (Figure 6c, Figure S9). The cryoTEM images confirm that in the case of R6 nanogel, a poorly defined structure with a broad diameter distribution was created (Figure 6f, Figure S9). 

The sizes of the structures visible on AFM images also confirmed the results obtained by DLS (Figure S10).

## 4. Conclusions

The research carried out here was intended to prepare thermoresponsive nanogels, based on crosslinked mesoglobules, which can be used in the future as drug carriers. The nanogels were based on modified copolymers of di(ethylene glycol) methyl ether methacrylate and 2-aminoethyl methacrylate. The aim was to verify how the numbers of functional groups affect the thermoresponsivity of the copolymers, their aggregation in water, and the size and stability of nanogels obtained from these copolymers. We have shown that as the amount of comonomer A in the copolymer chain increases, the resulting copolymers have a higher phase transition temperature. After the modification of amine groups with azide and propargyl groups, the T_CP_ of obtained polymers decreased, especially in the case of copolymers with propargyl groups. For the P(D-co-A) and P(D-co-N_3_) series, a low number of reactive functional groups and a high copolymer concentration are necessary to induce clear temperature responsiveness. 

The obtained copolymers formed nanoparticles whose sizes, besides the concentration of polymer solutions and heating rate, were influenced by the kind and number of functional groups in a copolymer. The sizes of obtained P(D-co-N_3_) and P(D-co-Prop) particles decreased when the numbers of functional groups in the chain increased. Aggregation measurements showed that polymers that had about 21 functional groups per chain formed smaller particles than those with only five functional groups per chain. This may be related to the degree of particle density and the possibility of the interaction with water. 

Huisgen’s cycloaddition reaction of a binary mixture of copolymers was very helpful in obtaining stable thermoresponsive nanogels. Both the sizes of nanogels and their degrees of swelling were dependent on the number of crosslinked groups and free, unreacted functional groups. When the amount of the crosslinked group increased, smaller particles with significantly lower swelling ratios were obtained. The ratio of the number of crosslinking groups also influenced the formation of gels. Nanogels composed from copolymers with significantly different amounts of functional groups created inhomogeneous particles that could crosslink intermolecularly and could finally form complex structures with larger sizes and irregular shapes. The obtained thermoresponsive nanogels were stable both above and below the transition temperature of the polymers from which they were made.

## Figures and Tables

**Table 1:** Characteristic of the P(D-co-A) copolymers prepared by ATRP.

Copolymer	Molar Ratio D:A ^1^	M_theor._ [g/mol] ^2^	D:A Conversion [%] ^3^	DP from Conversion [D:A] ^3^	M_n,conv_ [g/mol] ^4^	M_n_ [g/mol] ^5^	M_w_/M_n_^5^	Amount of A via Kaiser Test	
								[%mol]	Per Chain
P(D-co-A)_1	276:24	55 800	59:58	163:14	33 300	32 900	1.10	3	5
P(D-co-A)_2	270:30	55 700	56:60	151:18	32 000	34 900	1.15	8	17
P(D-co-A)_3	255:45	55 400	40:42	102:19	23 000	34 400	1.12	10	21
P(D-co-A)_4	240:60	55 100	50:45	120:27	28 000	41 000	1.14	24	60
P(D-co-A)_5	225:75	54 700	50:42	113:31	27 000	30 500	1.14	35	66

^1^ molar ratio of D to A in the polymerization mixture, ^2^ molar masses calculated from the mole ratio of comonomers to initiator in the polymerization mixture, ^3^ measured by HPLC, ^4^ calculated from M_n_,conv. = conv × ([M]0/[I]0) × M_monomer_ + M_initiator_, ^5^ measured by GPC-MALLS in DMF.

**Table 2:** Cloud point temperatures for water solutions of P(D-co-A), P (D-co-N_3_), and (P(D-co-Prop) at chosen copolymer concentration of 1.0 g/L, determined via UV-Vis and DLS measurements.

Amount of A in Initial P(D-co-A)		T_CP_ [°C]					
[%mol]	Per Chain	P(D-co-A)		P(D-co-N_3_)		P(D-co-Prop)	
		UV-Vis	DLS	UV-Vis	DLS	UV-Vis	DLS
3	5	32.2	36.6	31.8	30.7	16.6	17.9
8	17	35.0	42.7	34.4	29.7	13.6	15.8
10	21	37.3	43.0	-^1^	38.4	11.6	11.2
24	60	42.6	47.8	-^1^	-^1^	-^2^	-^2^
35	66	48.0	48.5	-^1^	-^1^	-^2^	-^2^

^1^ transmittance dropped only to 98%, ^2^ transition temperature too low for measurement.

**Table 3:** Average apparent hydrodynamic radii $R_{h}^{90}$ of mesoglobules formed by copolymers at 0.5 g/L above the T_CP_ (obtained by gradual heating)

Amount of A in Initial P(D-co-A)		$R_{h}^{90}\;[\mathrm{nm}]$		
[%mol]	Per Chain	P(D-co-A)	P(D-co-N_3_)	P(D-co-Prop)
3	5	120	132	599
8	17	77	118	158
10	21	79	76	68
24	60	-^1^	-^1^	-^2^
35	66	-^1^	-^1^	-^2^

^1^ No particles were detected, ^2^ No particles were detected, as copolymer was not dissolved at this condition.

**Table 4:** Hydrodynamic radii of crosslinked nanoparticles of mixed copolymers at 0.5 g/L (abrupt heating).

				$R_{h}^{90\;}[\mathrm{nm}]/(\mathrm{PDI})$						Swelling Ratio V_10_/V_60_^4^
				Before Reaction		After Reaction				
Polymer Mixture	Amount of Functional Groups Per Chain			60 °C		60 °C		10 °C		
	N_3_^1^	Prop ^1^	N_3_^2^							
R1	5	5	0	44.0	0.037	44.5	0.054	60.5	0.133	2.5
R2	17	17	0	41.0	0.116	42.5	0.299	51.0	0.018	1.7
R3	21	21	0	26/91.0	0.237 ^3^	42/167	0.152 ^3^	219.5	0.199	2.3
R4	21	17	4	21.5	0.300	25.5	0.145	36.0	0.269	2.8
R5	17	5	12	44.0	0.190	47.5	0.187	68.5	0.244	3.0
R6	21	5	16	30/89.5	0.332 ^3^	151.5	0.100	176.5	0.084	1.6

^1^ amount of N_3_ and Prop in copolymers used in crosslinking reaction, ^2^ amount of N_3_ that remained free after the crosslinking reaction, ^3^ PDI measured for dominating fraction, ^4^ V_10_/V_60_ the swelling ratio calculated as the ratio of hydrodynamic volumes of the nanogels at 10 and 60 °C

## Supplementary Materials

The following are available online at https://www.mdpi.com/2073-4360/12/8/1645/s1, Figure S1: Schematic illustration of synthesis of (a) P(D-co-A) copolymer, (b) reaction of P(D-co-A) with 2-azido-1,3-dimethylimidazolinium hexafluorophosphate, and (c) reaction of P(D-co-A) with propargyl chloroformate, Figure S2: Absorbance of P(D-co-A) copolymers before and after modifications, Figure S3: An exemplary ^1^H NMR spectra of (a) P(D-co-A)_3, (b) P(D-co-N_3_)_3, (c) P(D-co-Prop)_3 (600 MHz, in CDCl_3_), Figure S4: An exemplary FTIR spectra of (a) P(D-co-A)_3, (b) P(D-co-N3)_3, (c) P(D-co-Prop)_3, Figure S5: Transmittance curves of (a) P(D-co-A)_1, (b) P(D-co-A)_2, (c) P(D-co-A)_3, (d) P(D-co-A)_4 and (e) P(D-co-A)_5 copolymers at different solution concentrations, Figure S6: Transmittance curves of (a) P(D-co-N_3_)_1, (b) P(D-co-N_3_)_2, (c) P(D-co-N_3_)_3, (d) P(D-co-N_3_)_4 and (e) P(D-co-N_3_)_5 copolymers at different solution concentrations, Figure S7: Transmittance curves of (a) P(D-co-Prop)_1, (b) P(D-co-Prop)_2 and (c) P(D-co-Prop)_3 copolymers at different solution concentrations, Figure S8: Size distributions of the nanoparticles before and after crosslinking reaction formed in binary aqueous solutions of (a) P(D-co-N_3_)_3/P(D-co-Prop)_3 (R3) and (b) P(D-co-N_3_)_3/P(D-co-Prop)_1 (R6), Figure S9: Histograms of average diameter of nanogels calculated based on 100 particles measurements, Figure S10: An AFM images and their cross sections for R1, R3, R4 and R6 nanogels, Table S1: Cloud point temperatures for water solutions of P(D-co-A), P(D-co-N_3_) and P(D-co-Prop) copolymers at different solution concentrations, Table S2: Hydrodynamic radius $R_{h}^{90}$ of particles at different solution concentrations.

## References

[1] Soni K.S., Desale S.S., Bronich T.K. (2016). Nanogels: An overview of properties, biomedical applications and obstacles to clinical translation. J. Control. Release.

[2] Yadav H.K., Halabi N.A.A., Alsalloum G.A. (2017). Nanogels as novel drug delivery systems—A review. J. Pharm. Pharm. Res..

[3] Neamtu I., Rusu A.G., Diaconu A., Nita L.E., Chiriac A.P. (2017). Basic concepts and recent advances in nanogels as carriers for medical applications. Drug Deliv..

[4] Sabir F., Asad M.I., Qindeel M., Afzal I., Dar M.J., Shah K.U., Zeb A., Khan G.M., Ahmed N., Din F.-U. (2019). Polymeric nanogels as versatile nanoplatforms for biomedical applications. J. Nanomater..

[5] Paul S.D., Sharma H., Jeswani G., Jha A.K., Andronescu E., Grumezescu A.M. (2017). Chapter 12—Novel gels: Implications for drug delivery. Nanostructures for Drug Delivery.

[6] Nagel G., Sousa-Herves A., Wedepohl S., Calderón M. (2020). Matrix metalloproteinase-sensitive multistage nanogels promote drug transport in 3d tumor model. Theranostics.

[7] Navarro L., Theune L.E., Calderón M. (2020). Effect of crosslinking density on thermoresponsive nanogels: A study on the size control and the kinetics release of biomacromolecules. Eur. Polym. J..

[8] Gao L., Zabihi F., Ehrmann S., Hedtrich S., Haag R. (2019). Supramolecular nanogels fabricated via host–guest molecular recognition as penetration enhancer for dermal drug delivery. J. Control. Release.

[9] Dey P., Bergmann T., Cuellar-Camacho J.L., Ehrmann S., Chowdhury M.S., Zhang M., Dahmani I., Haag R., Azab W. (2018). Multivalent flexible nanogels exhibit broad-spectrum antiviral activity by blocking virus entry. ACS Nano.

[10] Aseyev V., Tenhu H., Winnik F.M., Müller H.E.A., Borisov O. (2011). Non-ionic thermoresponsive polymers in water. Self Organized Nanostructures of Amphiphilic Block Copolymers II.

[11] Aseyev V.O., Tenhu H., Winnik F.M., Khokhlov A.R. (2006). Temperature dependence of the colloidal stability of neutral amphiphilic polymers in water. Conformation-Dependent Design of Sequences in Copolymers II.

[12] Dawson K.A., Gorelov A.V., Timoshenko E.G., Kuznetsov Y.A., Du Chesne A. (1997). Formation of mesoglobules from phase separation in dilute polymer solutions: A study in experiment, theory, and applications. Phys. A Stat. Mech. Appl..

[13] Gorelov A.V., Du Chesne A., Dawson K.A. (1997). Phase separation in dilute solutions of poly (N-isopropylacrylamide). Phys. A Stat. Mech. Appl..

[14] Plummer R., Hill D.J.T., Whittaker A.K. (2006). Solution properties of star and linear poly(N-isopropylacrylamide). Macromolecules.

[15] Xu J., Liu S. (2009). Synthesis of well-defined 7-arm and 21-arm poly(N-isopropylacrylamide) star polymers with β-cyclodextrin cores via click chemistry and their thermal phase transition behavior in aqueous solution. J. Polym. Sci. A Polym. Chem..

[16] Yuan W., Yuan J., Zhou M., Sui X. (2006). Synthesis, characterization, and thermal properties of dendrimer-star, block-comb copolymers by ring-opening polymerization and atom transfer radical polymerization. J. Polym. Sci. A Polym. Chem..

[17] Lambermont-Thijs H.M.L., Hoogenboom R., Fustin C.-A., Bomal-D’Haese C., Gohy J.-F., Schubert U.S. (2009). Solubility behavior of amphiphilic block and random copolymers based on 2-ethyl-2-oxazoline and 2-nonyl-2-oxazoline in binary water–ethanol mixtures. J. Polym. Sci. A Polym. Chem..

[18] Seno K.-I., Tsujimoto I., Kikuchi T., Kanaoka S., Aoshima S. (2008). Thermosensitive gradient copolymers by living cationic polymerization: Semibatch precision synthesis and stepwise dehydration-induced micellization and physical gelation. J. Polym. Sci. A Polym. Chem..

[19] Meeussen F., Nies E., Berghmans H., Verbrugghe S., Goethals E., Du Prez F. (2000). Phase behaviour of poly(N-vinyl caprolactam) in water. Polymer.

[20] Van Durme K., Van Assche G., Van Mele B. (2004). Kinetics of demixing and remixing in poly(N-isopropylacrylamide)/water studied by modulated temperature dsc. Macromolecules.

[21] Zhang Y., Cremer P.S. (2006). Interactions between macromolecules and ions: The hofmeister series. Curr. Opin. Chem. Biol..

[22] Zhang Y., Furyk S., Bergbreiter D.E., Cremer P.S. (2005). Specific ion effects on the water solubility of macromolecules:  Pnipam and the hofmeister series. J. Am. Chem. Soc..

[23] Laschewsky A., Rekaï E.D. (2000). Photochemical modification of the lower critical solution temperature of cinnamoylated poly(N-2-hydroxypropylmethacrylamide) in water. Macromol. Rapid Commun..

[24] Trzebicka B., Weda P., Utrata-Wesołek A., Dworak A., Tsvetanov C. (2010). Mesoglobules of random copolyethers as templates for nanoparticles. J. Polym. Sci. A Polym. Chem..

[25] Dworak A., Lipowska D., Szweda D., Suwinski J., Trzebicka B., Szweda R. (2015). Degradable polymeric nanoparticles by aggregation of thermoresponsive polymers and “click” chemistry. Nanoscale.

[26] Lutz J.-F. (2008). Polymerization of oligo(ethylene glycol) (meth)acrylates: Toward new generations of smart biocompatible materials. J. Polym. Sci. A Polym. Chem..

[27] Lutz J.-F., Weichenhan K., Akdemir Ö., Hoth A. (2007). About the phase transitions in aqueous solutions of thermoresponsive copolymers and hydrogels based on 2-(2-methoxyethoxy)ethyl methacrylate and oligo(ethylene glycol) methacrylate. Macromolecules.

[28] Siu M., He C., Wu C. (2003). Formation of mesoglobular phase of amphiphilic copolymer chains in dilute solution:  Effect of comonomer distribution. Macromolecules.

[29] Agarwal S., Kumar R., Kissel T., Reul R. (2009). Synthesis of degradable materials based on caprolactone and vinyl acetate units using radical chemistry. Polym. J..

[30] Jamróz-Piegza M., Utrata-Wesołek A., Trzebicka B., Dworak A. (2006). Hydrophobic modification of high molar mass polyglycidol to thermosensitive polymers. Eur. Polym. J..

[31] Jerca F.A., Anghelache A.M., Ghibu E., Cecoltan S., Stancu I.-C., Trusca R., Vasile E., Teodorescu M., Vuluga D.M., Hoogenboom R. (2018). Poly(2-isopropenyl-2-oxazoline) hydrogels for biomedical applications. Chem. Mater..

[32] Jerca F.A., Jerca V.V., Anghelache A.M., Vuluga D.M., Hoogenboom R. (2018). Poly(2-isopropenyl-2-oxazoline) as a versatile platform towards thermoresponsive copolymers. Polym. Chem..

[33] Zhang G., Wu C., Khokhlov A.R. (2006). Folding and formation of mesoglobules in dilute copolymer solutions. Conformation-Dependent Design of Sequences in Copolymers I.

[34] Aseyev V., Hietala S., Laukkanen A., Nuopponen M., Confortini O., Du Prez F.E., Tenhu H. (2005). Mesoglobules of thermoresponsive polymers in dilute aqueous solutions above the LCST. Polymer.

[35] Rangelov S., Simon P., Toncheva-Moncheva N., Dimitrov P., Gajewska B., Tsvetanov C.B. (2012). Nanosized colloidal particles from thermosensitive poly(methoxydiethyleneglycol methacrylate)s in aqueous media. Polym. Bull..

[36] Trzebicka B., Szweda D., Rangelov S., Kowalczuk A., Mendrek B., Utrata-Wesołek A., Dworak A. (2013). (Co)polymers of oligo(ethylene glycol) methacrylates—temperature-induced aggregation in aqueous solution. J. Polym. Sci. A Polym. Chem..

[37] Wu C., Li W., Zhu X.X. (2004). Viscoelastic effect on the formation of mesoglobular phase in dilute solutions. Macromolecules.

[38] Junk M.J.N., Li W., Schlüter A.D., Wegner G., Spiess H.W., Zhang A., Hinderberger D. (2011). Formation of a mesoscopic skin barrier in mesoglobules of thermoresponsive polymers. J. Am. Chem. Soc..

[39] Kujawa P., Aseyev V., Tenhu H., Winnik F.M. (2006). Temperature-sensitive properties of poly(N-isopropylacrylamide) mesoglobules formed in dilute aqueous solutions heated above their demixing point. Macromolecules.

[40] Barker I.C., Cowie J.M.G., Huckerby T.N., Shaw D.A., Soutar I., Swanson L. (2003). Studies of the “smart” thermoresponsive behavior of copolymers of N-isopropylacrylamide and N,N-dimethylacrylamide in dilute aqueous solution. Macromolecules.

[41] Nuopponen M., Ojala J., Tenhu H. (2004). Aggregation behaviour of well defined amphiphilic diblock copolymers with poly(N-isopropylacrylamide) and hydrophobic blocks. Polymer.

[42] Toncheva-Moncheva N., Dimitrov P., Tsvetanov C.B., Robak B., Trzebicka B., Dworak A., Rangelov S. (2011). Formation of mesoglobules in aqueous media from thermo-sensitive poly(ethoxytriethyleneglycol acrylate). Polym. Bull..

[43] Lipowska-Kur D., Szweda R., Trzebicka B., Dworak A. (2018). Preparation and characterization of doxorubicin nanocarriers based on thermoresponsive oligo(ethylene glycol) methyl ether methacrylate polymer-drug conjugates. Eur. Polym. J..

[44] Libera M., Trzebicka B., Kowalczuk A., Wałach W., Dworak A. (2011). Synthesis and thermoresponsive properties of four arm, amphiphilic poly(tert-butyl-glycidylether)-block-polyglycidol stars. Polymer.

[45] Libera M., Wałach W., Trzebicka B., Rangelov S., Dworak A. (2011). Thermosensitive dendritic stars of tert-butyl-glycidylether and glycidol—Synthesis and encapsulation properties. Polymer.

[46] Haladjova E., Rangelov S., Tsvetanov C., Simon P. (2014). Preparation of polymeric nanocapsules via nano-sized poly(methoxydiethyleneglycol methacrylate) colloidal templates. Polymer.

[47] Haladjova E., Simeonova M., Rangelov S., Tsvetanov C., Lalev G. (2016). Template-assisted approach for preparation of poly(butyl-2-cyanoacrylate) nanocapsules. Nanosci. Nanotechnol..

[48] Ivanova T., Haladjova E., Mees M., Momekova D., Rangelov S., Momekov G., Hoogenboom R. (2016). Characterization of polymer vector systems based on partially hydrolyzed polyoxazoline for gene transfection. Pharmacia.

[49] Trzcinska R., Szweda D., Rangelov S., Suder P., Silberring J., Dworak A., Trzebicka B. (2012). Bioactive mesoglobules of poly(di(ethylene glycol) monomethyl ether methacrylate)–peptide conjugate. J. Polym. Sci. A Polym. Chem..

[50] Trzebicka B., Haladjova E., Otulakowski Ł., Oleszko N., Wałach W., Libera M., Rangelov S., Dworak A. (2015). Hybrid nanoparticles obtained from mixed mesoglobules. Polymer.

[51] Trzebicka B., Robak B., Trzcinska R., Szweda D., Suder P., Silberring J., Dworak A. (2013). Thermosensitive pnipam-peptide conjugate—Synthesis and aggregation. Eur. Polym. J..

[52] Weda P., Trzebicka B., Dworak A., Tsvetanov C.B. (2008). Thermosensitive nanospheres of low-density core—An approach to hollow nanoparticles. Polymer.

[53] Osváth Z., Iván B. (2017). The dependence of the cloud point, clearing point, and hysteresis of poly(N-isopropylacrylamide) on experimental conditions: The need for standardization of thermoresponsive transition determinations. Macromol. Chem. Phys..

[54] Virtanen J., Tenhu H. (2000). Thermal properties of poly(N-isopropylacrylamide)-g-poly(ethylene oxide) in aqueous solutions: Influence of the number and distribution of the grafts. Macromolecules.

[55] Xu X.D., Chen C.S., Wang Z.C., Wang G.R., Cheng S.X., Zhang X.Z., Zhuo R.X. (2008). “Click” chemistry for in situ formation of thermoresponsive p(NIPAM-co-HEMA)-based hydrogels. J. Polym. Sci. A Polym. Chem..

[56] Vasilevskaya V.V., Khalatur P.G., Khokhlov A.R. (2003). Conformational polymorphism of amphiphilic polymers in a poor solvent. Macromolecules.

