# Supplementary material for: Thermoresponsive Nanogels of Modified Poly((di(ethylene glycol) methyl ether methacrylate)-co-(2-aminoethyl methacrylate))s
Source: Polymers (Basel). 2020 Jul 24;12(8):1645. doi: 10.3390/polym12081645 (PMC7463910; doi:10.3390/polym12081645)
Supplement: Supplementary file 1 [file polymers-12-01645-s001.pdf]

Electronic Supplementary Information

Thermoresponsive nanogels of modified poly((di(ethylene glycol) methyl ether methacrylate)-co-(2-aminoethyl methacrylate))s

D. Lipowska-Kur, Ł. Otulakowski, B. Trzebicka, A. Utrata-Wesołek, A. Dworak*

Table of contents

Figure S1. Schematic illustration of synthesis of a) P(D-co-A) copolymer, b) reaction of P(D-co-A) with 2-azido-1,3-dimethylimidazolinium hexafluorophosphate, and c) reaction of P(D-co-A) with propargyl chloroformate.

Figure S2. Absorbance of P(D-co-A) copolymers before and after modifications.

Figure S3. An exemplary ^{1}H NMR spectra of a) P(D-co-A)_3, b) P(D-co-N_3)_3, c) P(D-co-Prop)_3 (600 MHz, in $CDCl_3$).

Figure S4. An exemplary FTIR spectra of a) P(D-co-A)_3, b) P(D-co-N3)_3, c) P(D-co-Prop)_3.

Figure S5. Transmittance curves of a) P(D-co-A)_1, b) P(D-co-A)_2, c) P(D-co-A)_3, d) P(D-co-A)_4 and e) P(D-co-A)_5 copolymers at different solution concentrations.

Figure S6. Transmittance curves of a) P(D-co-N_3)_1, b) P(D-co-N_3)_2, c) P(D-co-N_3)_3, d) P(D-co-N_3)_4 and e) P(D-co-N_3)_5 copolymers at different solution concentrations.

Figure S7. Transmittance curves of a) P(D-co-Prop)_1, b) P(D-co-Prop)_2 and c) P(D-co-Prop)_3 copolymers at different solution concentrations.

Figure S8. Size distributions of the nanoparticles before and after crosslinking reaction formed in binary aqueous solutions of a) P(D-co-N_3)_3/P(D-co-Prop)_3 (R3) and b) P(D-co-N_3)_3/P(D-co-Prop)_1 (R6).

Figure S9. Histograms of average diameter of nanogels calculated based on 100 particles measurements

Figure S10. An AFM images and their cross sections for R1, R3, R4 and R6 nanogels.

Table S1. Cloud point temperatures for water solutions of P(D-co-A), P(D-co-N_3) and P(D-co-Prop) copolymers at different solution concentrations.

Table S2. Hydrodynamic radius R_h^{90} of particles at different solution concentrations.

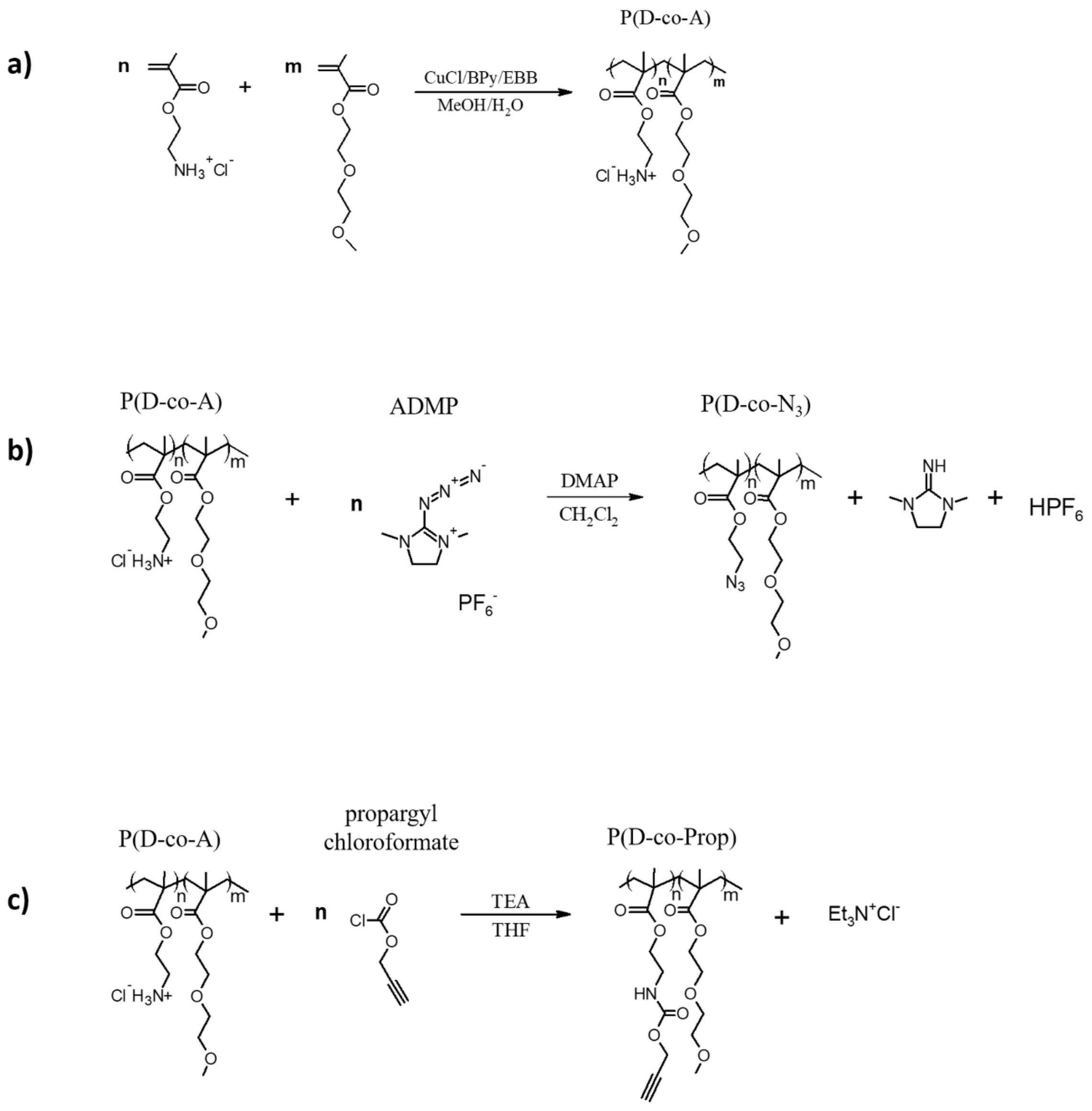

Figure S1. Schematic illustration of synthesis of a) P(D-co-A) copolymer, b) reaction of P(D-co-A) with 2-azido-1,3-dimethylimidazolinium hexafluorophosphate, and c) reaction of P(D-co-A) with propargyl chloroformate.

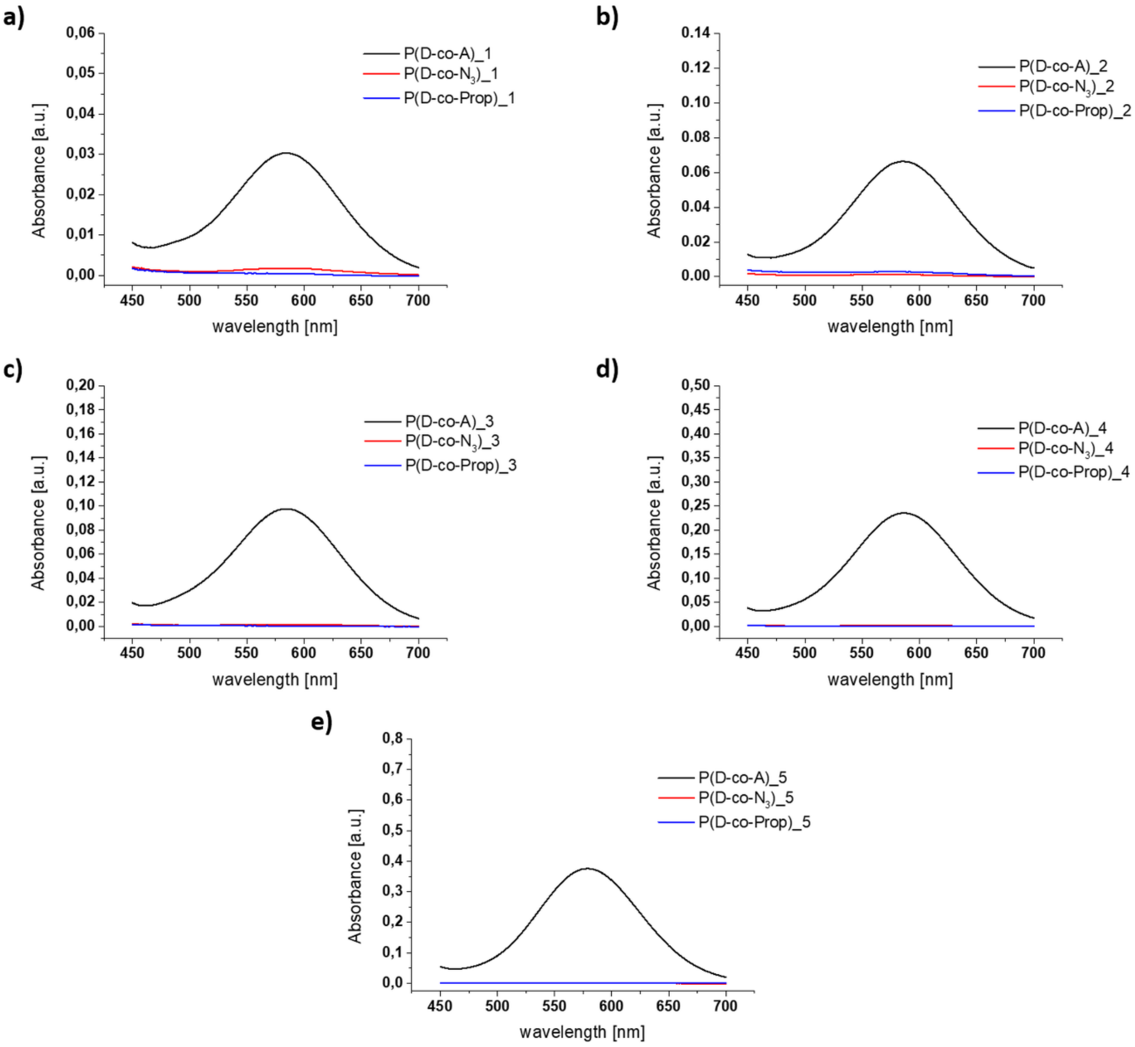

Figure S2. Absorbance of P(D-co-A) copolymers before and after modifications.

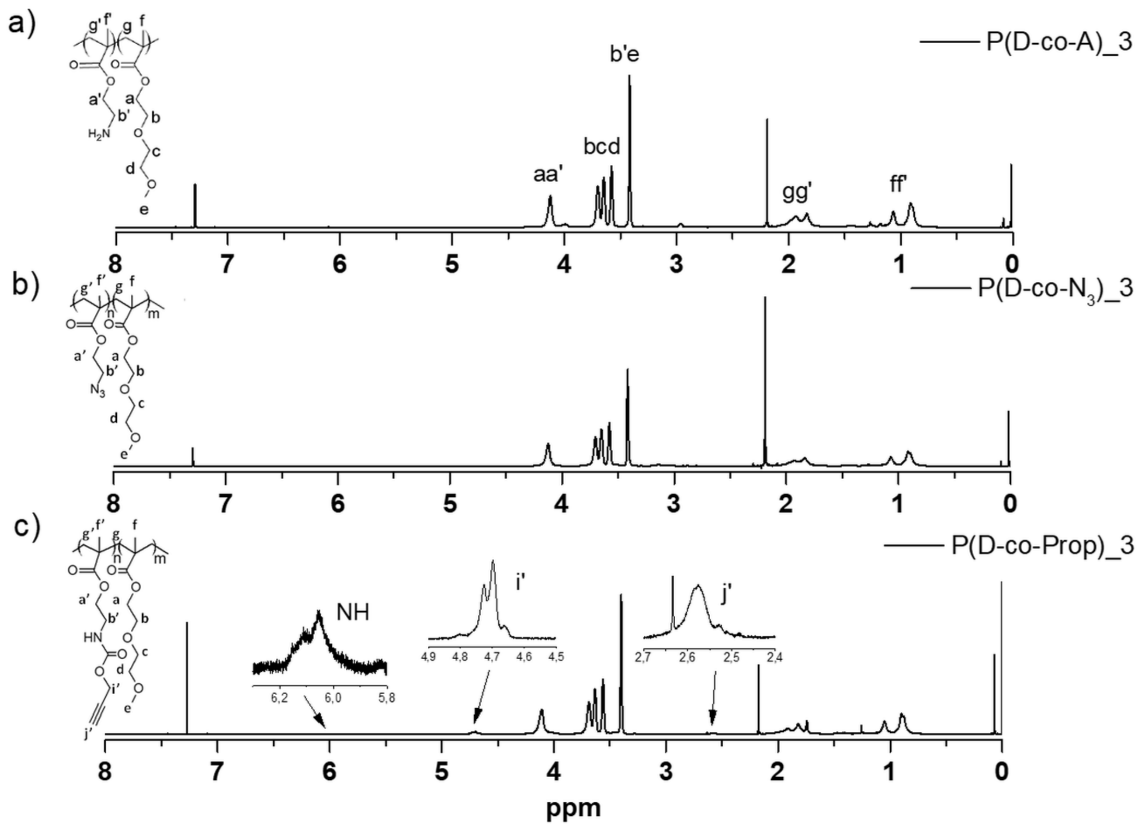

Figure S3. An exemplary ^{1}H NMR spectra of a) P(D-co-A)_3, b) P(D-co-N_3)_3, c) P(D-co-Prop)_3 (600 MHz, in $CDCl_3$).

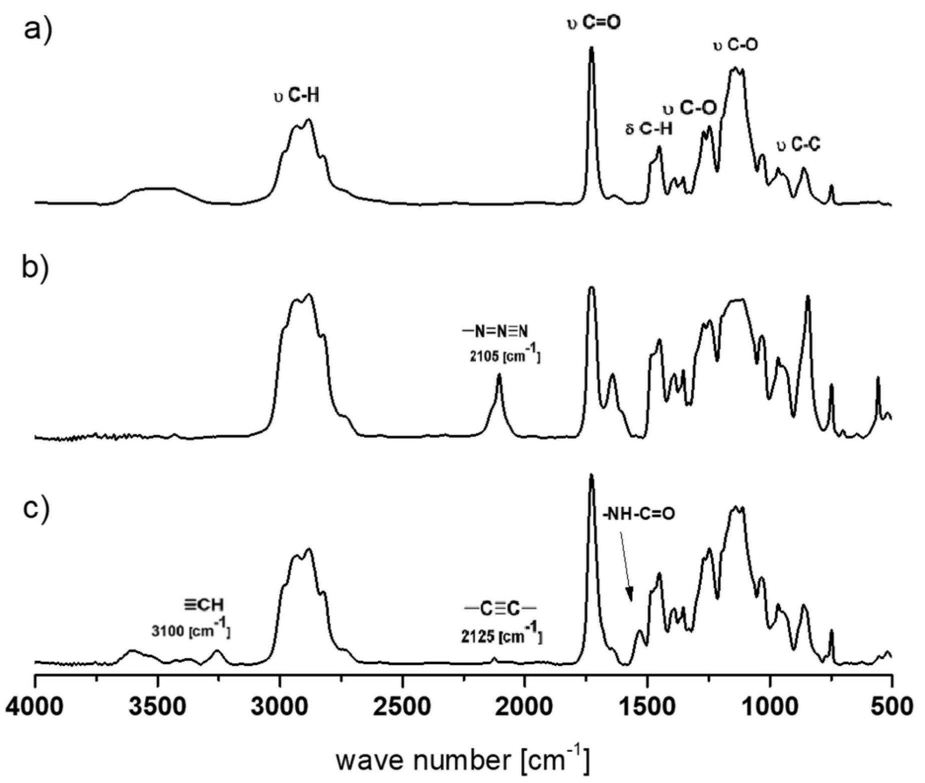

Figure S4. An exemplary FTIR spectra of a) P(D-co-A)_3, b) P(D-co-N_3)_3, c) P(D-co-Prop)_3.

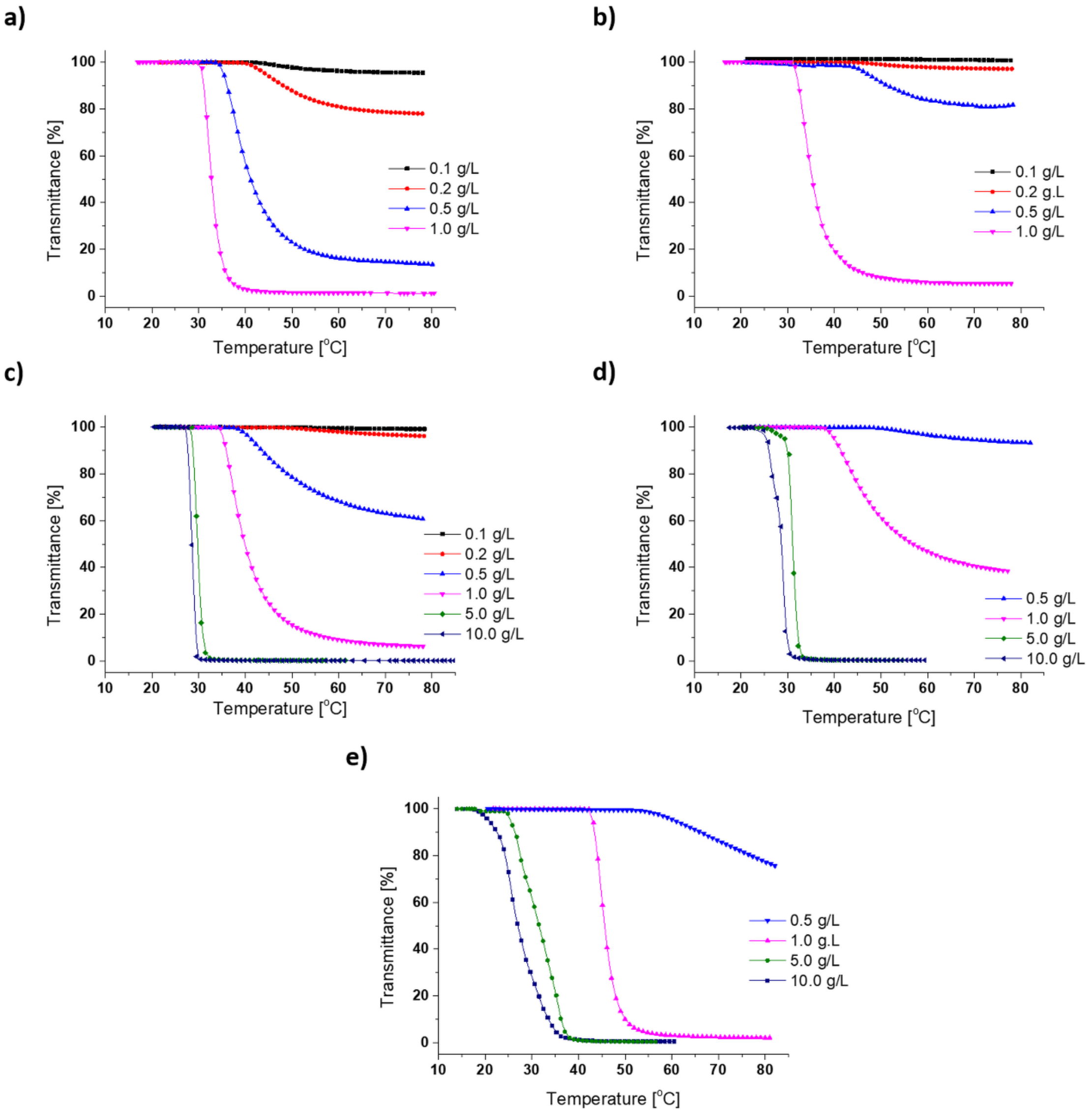

Figure S5. Transmittance curves of a) P(D-co-A)_1, b) P(D-co-A)_2, c) P(D-co-A)_3, d) P(D-co-A)_4 and e) P(D-co-A)_5 copolymers at different solution concentrations.

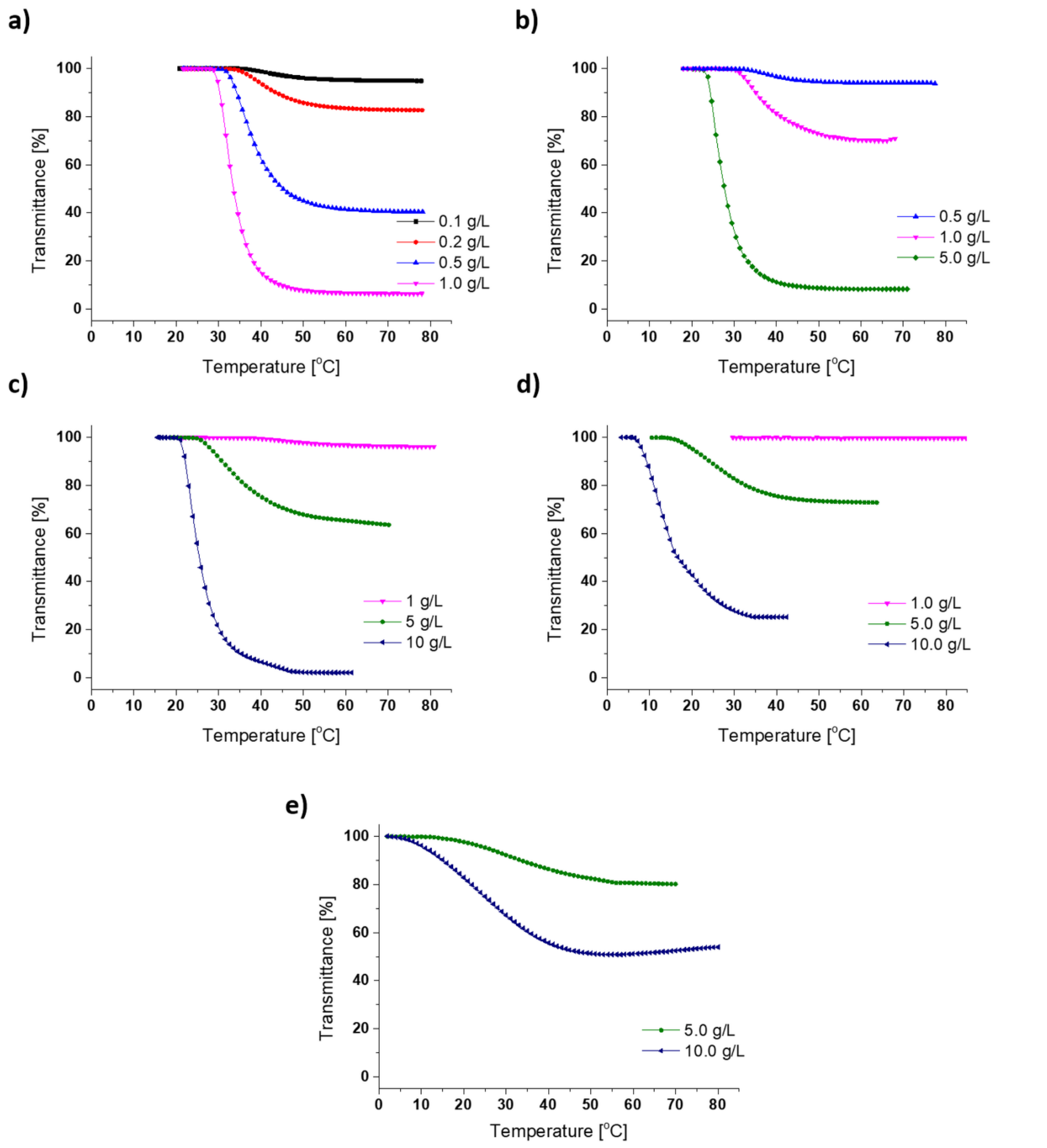

Figure S6. Transmittance curves of a) P(D-co-N_3)_1, b) P(D-co-N_3)_2, c) P(D-co-N_3)_3, d) P(D-co-N_3)_4 and e) P(D-co-N_3)_5 copolymers at different solution concentrations.

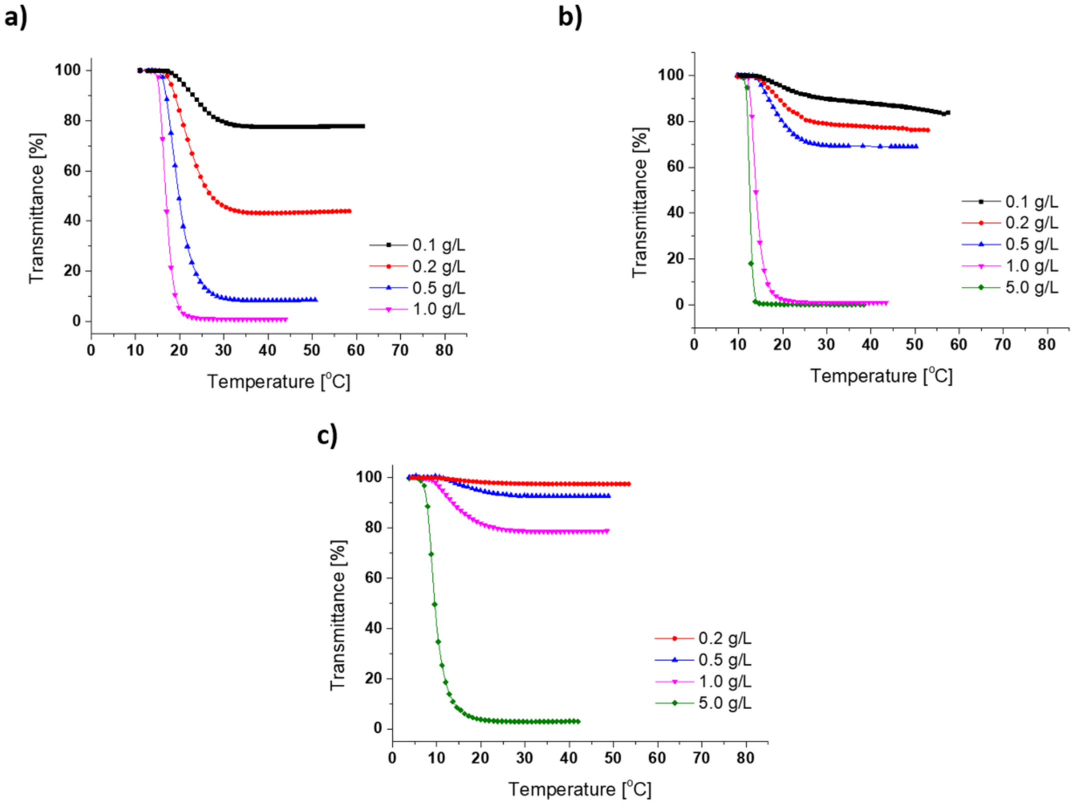

Figure S7. Transmittance curves of a) P(D-co-Prop)_1, b) P(D-co-Prop)_2 and c) P(D-co-Prop)_3 copolymers at different solution concentrations.

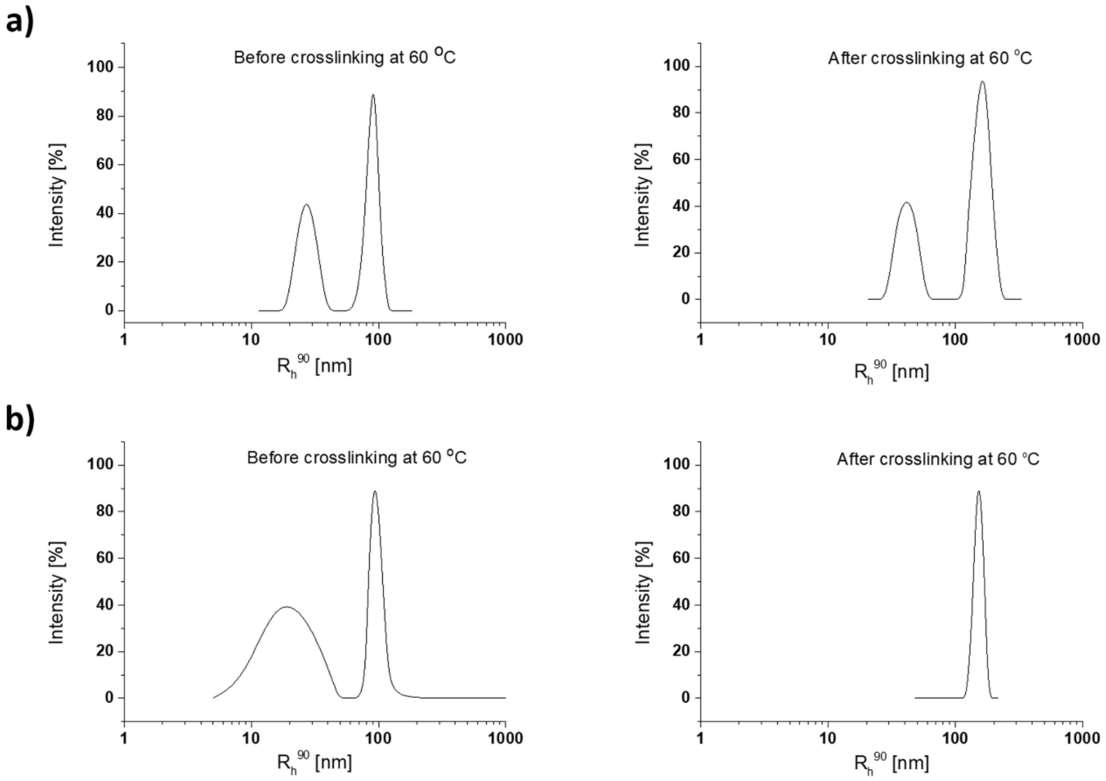

Figure S8. Size distributions of the nanoparticles before and after crosslinking reaction formed in binary aqueous solutions of a) P(D-co-N_3)_3/P(D-co-Prop)_3 (R3) and b) P(D-co-N_3)_3/P(D-co-Prop)_1 (R6).

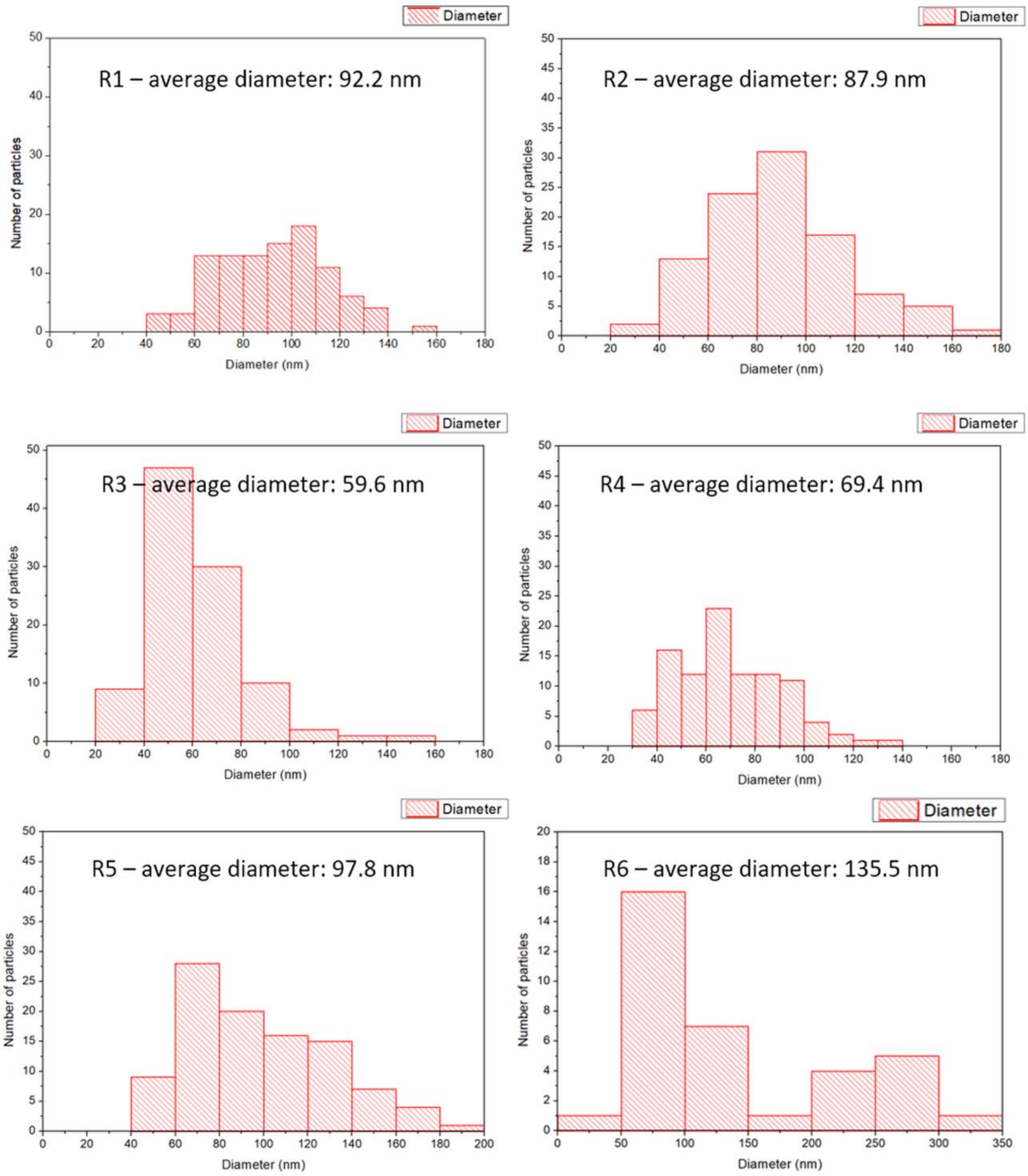

Figure S9. Histograms of average diameter of nanogels calculated based on 100 particles measurements.

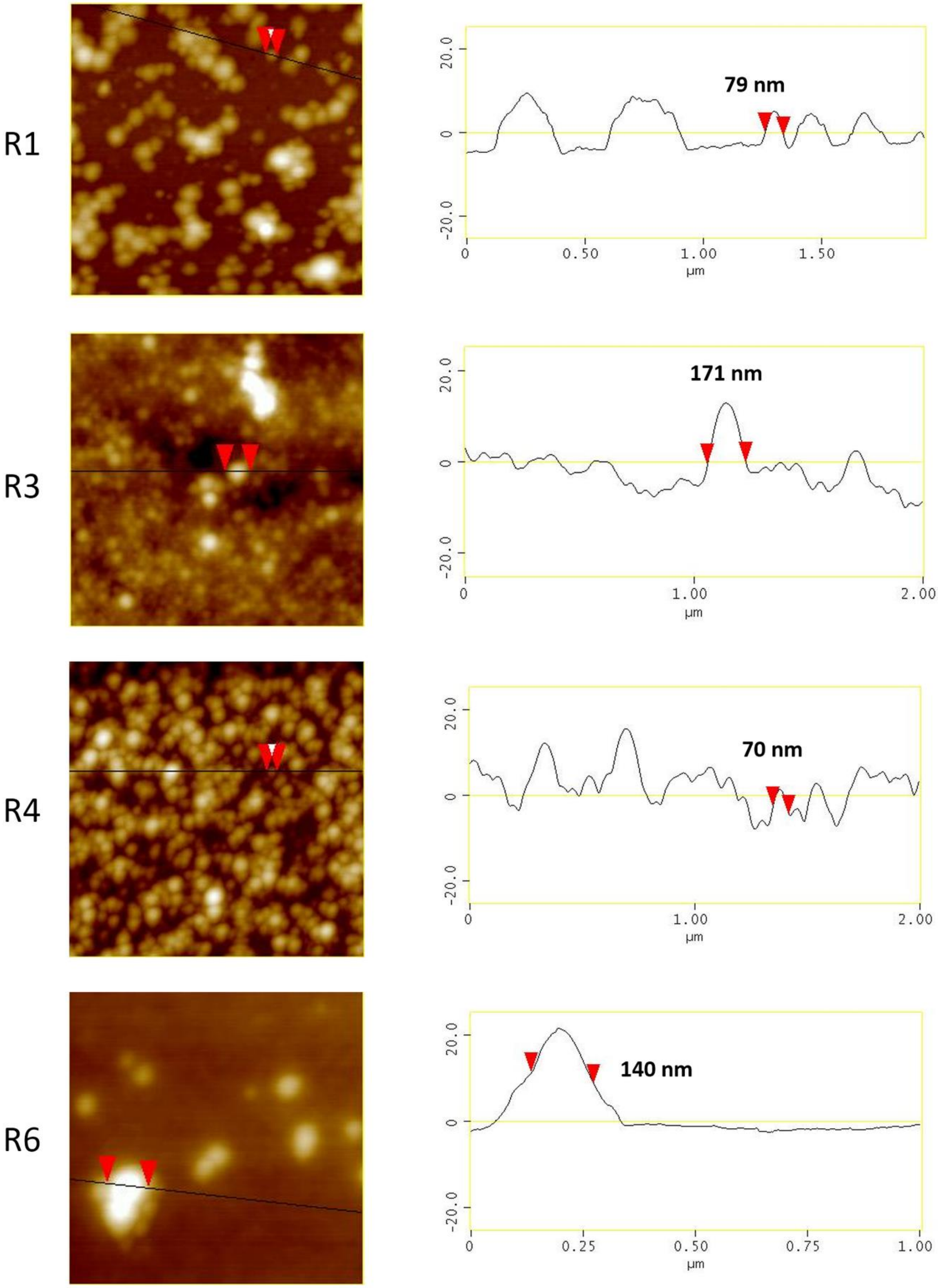

Figure S10. An AFM images and their cross sections for R1, R3, R4 and R6 nanogels

Table S1. Cloud point temperatures for water solutions of P(D-co-A), P(D-co-N_3) and P(D-co-Prop) copolymers at different solution concentrations

Amount of A in initial P(D-co-A)		C [g/L]	T_{CP} [°C]					
[% mol]	Per chain		P(D-co-A)		P(D-co-N_3)		P(D-co-Prop)	
			UV-Vis	DLS	UV-Vis	DLS	UV-Vis	DLS
3	5	1.0	32.2	36.6	31.8	30.7	16.6	17.9
		0.5	37.8	40.5	35.8	32.9	18.5	17.6
		0.2	45.0	44.3	39.8	35.8	20.4	19.8
		0.1	46.5	48.1	40.4	46.0	23.2	22.3
8	17	5.0	31.1	38.5	25.5	23.5	12.5	15.4
		1.0	35.0	42.7	34.4	29.7	13.6	15.8
		0.5	47.8	46.7	36.7	31.6	16.4	16.3
		0.2	48.4	49.4	-*	38.6	17.8	16.8
10	21	10	28.5	31.0	23.5	24.2	-^	-^
		5.0	29.7	31.0	26.8	26.5	9.0	8.9
		1.0	37.3	43.0	-*	38.4	11.6	11.2
		0.5	43.0	55.6	-*	45.9	13.4	11.5
		0.2	-*	61.7	-*	48.3	-*	12.1
24	60	10	28.9	27.9	19.2	17.2	-^	-^
		5.0	31.0	27.6	24.0	25.4	-^	-^
		1.0	42.6	47.8	-*	-*	-^	-^
		0.5	-*	-*	-*	-*	-^	-^
35	66	10	25.5	21.7	19.5	-^	-^	-^
		5.0	35.3	22.3	-*	-^	-^	-^
		1.0	48.0	48.5	-*	-*	-^	-^
		0.5	-*	-*	-*	-*	-^	-^

* transmittance dropped only to 98%

^ transition temperature too low for measurements

Table S2. Apparent hydrodynamic radii R_h^{90} of mesoglobules formed by copolymers at different solution concentrations obtained by gradual heating

Amount of A in initial P(D-co-A)		C [g/L]	R_h^{90} [nm]		
[%mol]	Per chain		P(D-co-A)	P(D-co-N_3)	P(D-co-Prop)
3	5	1.0	135	148	944
		0.5	120	132	599
		0.2	86	109	501
		0.1	78	75	92
8	17	5.0	150	113	291
		1.0	127	118	200
		0.5	77	118	158
		0.2	62	70	92
10	21	10	-#	-#	-^
		5.0	-#	-#	92
		1.0	111	108	70
		0.5	79	76	68
		0.2	43	48	55
24	60	10	-#	52	-^
		5.0	-#	43	-^
		1.0	84	-*	-^
		0.5	-*	-*	-^
35	66	10	-#	-^	-^
		5.0	128	-^	-^
		1.0	66	-*	-^
		0.5	-*	-*	-^

* No particles were detected
^ No particles were detected as copolymer was not dissolved at this condition
# DLS measurement above T_{CP} impossible, multiscattering is generated
